# Phase 1 Study of Oral N-Acetylmannosamine in Primary Podocytopathies

**DOI:** 10.1016/j.ekir.2025.103758

**Published:** 2025-12-31

**Authors:** Marjan Huizing, Anirban Ganguli, Jonathan Bolaños, Petcharat Leoyklang, Kenneth J. Wilkins, Yi Zeng, William D. Figg, Francis Rossignol, May Christine V. Malicdan, Jeffrey B. Kopp, William A. Gahl, NIH-ManNAc Study Team, Andrea Ashton, Jodi L. Blake, Thomas A. Cassini, Kevin J. O’Brien

**Affiliations:** 1 Medical Genetics Branch, National Human Genome Research Institute, National Institutes of Health, Bethesda, Maryland, USA; 2 Kidney Diseases Branch, National Institute of Diabetes and Digestive and Kidney Diseases, National Institutes of Health, Bethesda, Maryland, USA; 3 Biostatistics Program, Office of the Director, National Institute of Diabetes and Digestive and Kidney Diseases, National Institutes of Health, Bethesda, Maryland, USA; 4 Clinical Pharmacology Laboratory, National Institutes of Health Clinical Center, Bethesda, Maryland, USA

**Keywords:** N-acetylmannosamine (ManNAc), phase 1 trial, podocytopathy, proteinuria, sialylation

## Abstract

**Introduction:**

Terminal sialic acid (SA) residues on glycoconjugates are essential for maintaining the glomerular filtration barrier’s charge selectivity and podocyte ultrastructure. SA depletion affects key podocyte glycoproteins, contributing to podocytopathy and proteinuria. Glomerular hyposialylation is commonly seen in experimental podocytopathies and human renal biopsies. In nephrotic mouse models, oral administration of the metabolic SA precursor, N-acetylmannosamine (ManNAc) restored sialylation and reduced proteinuria, suggesting therapeutic potential.

**Methods:**

In this single-center, single-arm, ascending dose phase 1 trial, we evaluated safety and pharmacokinetics (PKs) of oral ManNAc in primary podocytopathies (ClinicalTrials.gov: NCT02639260). Eligible participants had urine protein-to-creatinine ratio (UPCR) > 1 g/g and estimated glomerular filtration rates (eGFR) > 15 ml/min per 1.73 m^2^. Six subjects received a single 3g ManNAc dose followed by 5 days of 1.5 g twice-daily (BID) dosing. One subject received a single 6 g dose.

**Results:**

All enrolled participants had primary podocytopathy, with eGFR of 25 to 89 ml/min per 1.73 m^2^ and UPCR of 1.1 to 9.21 g/g. ManNAc was well-tolerated without serious adverse events (AEs). Maximum plasma ManNAc concentration was reached within 2 to 4 hours postdose, with dose-dependent increases in plasma SA. Subjects with eGFR < 45 ml/min per 1.73 m^2^ showed elevated maximum plasma ManNAc concentration and area under curve for both ManNAc and SA, reflecting reduced renal clearance. Proteinuria reduction of 12% to 52% (regression-adjusted mean 9.69%, *P* < 0.0001) was observed in subjects receiving ManNAc BID, correlating with glomerular hyposialylation in pre-study renal biopsies.

**Conclusion:**

Oral ManNAc demonstrated short-term safety and increased plasma SA levels in podocytopathy subjects. Early efficacy signals suggest that proteinuria reduction may correlate with glomerular hyposialylation, identifying a potential treatment biomarker. A phase 2 trial (NCT06664814) is underway to assess long-term outcomes.

## Graphical abstract

**Figure:**
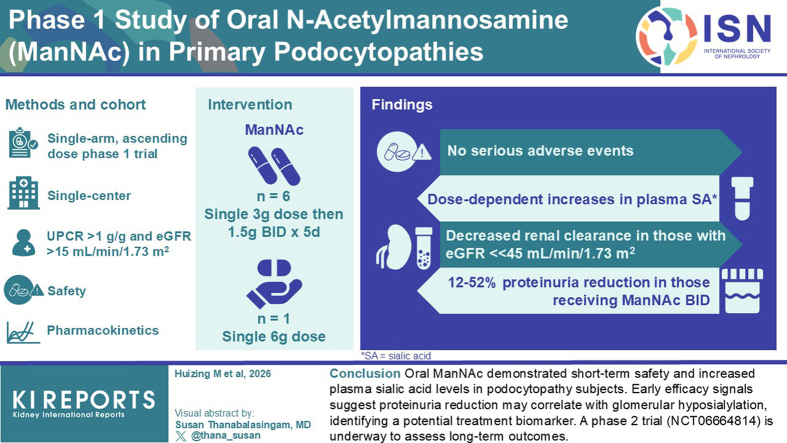

ManNAc is the biologic precursor of N-acetyl neuraminic acid (Neu5Ac), the most abundant SA in mammals.^1^ Terminal, negatively charged SA residues on glycoproteins and glycolipids (glycans) play crucial roles in glycocalyx integrity, oxidative stress protection, and cellular signaling.^2^, ^3^, ^4^, ^5^, ^6^, ^7^, ^8^, ^9^ Extracellular supplied ManNAc efficiently crosses cell-membranes and bypasses the cytoplasmic rate-limiting GNE enzyme, enhancing SA synthesis and glycan sialylation (Figure 1a).^2^^,^^3^ Altered sialylation has been implicated in certain malignancies, neurodegeneration, autoimmune disorders, myopathy, and renal disease.^5^^,^^10^, ^11^, ^12^, ^13^, ^14^ Lectin histochemistry has revealed glomerular hyposialylation in minimal change disease, focal segmental glomerulosclerosis (FSGS), membranous nephropathy, and IgA nephropathy, with a prevalence of up to 60%.^15^, ^16^, ^17^, ^18^, ^19^ Oxidative stress has been proposed as a contributing factor to reduced sialylation and the pathophysiology of renal disease.^5^^,^^14^^,^^20^, ^21^, ^22^ In subjects with impaired kidney function, reduced IgG sialylation was reported to enhance inflammation,^23^ aberrant IgA sialylation in IgA nephropathy was implied to disrupt podocyte architecture,^24^^,^^25^ and hyposialylation of IgM on T cells was suggested as a predictor of disease severity in children with nephrotic syndrome.^26^

**Figure 1:**
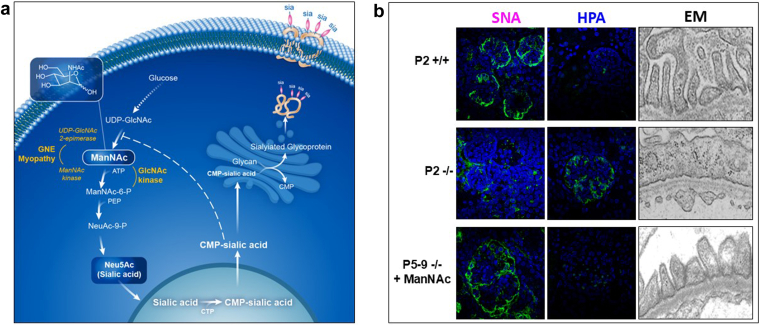
Preclinical data. (a) Intracellular Neu5Ac (sialic acid, SA) biosynthesis pathway. Cytosolic SA biosynthesis starts with the conversion of uridine diphosphate N-acetylglucosamine (UDP-GlcNAc, derived from glucose) to ManNAc and then to ManNAc-6P by the bifunctional enzyme UDP-GlcNAc 2-epimerase/ManNAc kinase (GNE, the enzyme deficient in GNE myopathy). ManNAc-6P undergoes 2 additional committed conversions to cytosolic free SA, which becomes activated to CMP-SA by CMP-SA synthase (CMAS) inside the nucleus. CMP-SA is a SA donor that sialylates ("sia") nascent glycans in the Golgi complex; it also regulates cytosolic SA synthesis by feedback inhibiting UDP-GlcNAc 2-epimerase activity through binding to its allosteric site (dashed line). Adapted, with permission from Xu *et al.*.^6^ (b) Effects of ManNAc therapy on lectin staining and ultrastructure of glomeruli in mice with SA synthesis deficiency, due to mutations in *Gne* (Gne^M712T/M712T^). Representative glomerular images stained with (green) fluorescent lectins SNA (binding to Neu5Ac), or HPA (binding to desialylated glycans terminating with GalNAc), and the DAPI nuclear dye (blue). Compared with wild-type mice (P2 +/+), mutant mice at postnatal day 2 (P2 -/-) showed severe hyposialylation by decreased SNA and increased HPA staining, with loss of the fenestrated podocyte ultrastructure in P2 -/- compared with +/+ littermates. ManNAc treatment rescued mutant mouse glomeruli from hyposialylation (P5-9 -/- + ManNAc), as demonstrated by a normal lectin staining pattern similar to that seen in wild-type mice (P2 +/+) and (partial) recovery of the fenestration of podocyte structure.^7^, ^8^, ^9^ ManNAc, N-acetylmannosamine; SA, sialic acid.

Animal models support a mechanistic link between glomerular hyposialylation and proteinuria. Sialidase injection^27^ and genetic deficiencies in the SA synthesis pathway (e.g., Cmas, Gne)^7^^,^^28^ induce podocytopathy and proteinuria in mice. Oral ManNAc restores podocyte structure and reduces proteinuria in Gne-deficient mice (Figure 1b).^7^, ^8^, ^9^^,^^29^ Similar benefits of ManNAc therapy are reported in aminonucleoside-induced nephrosis,^30^, ^31^, ^32^, ^33^ angiopoietin-like 4 overexpressing rat models of minimal change disease^34^ and streptozocin-induced diabetic nephropathy,^35^ implicating oxidative stress pathways.

ManNAc is under investigation for GNE myopathy, a rare genetic neuromuscular disorder with skeletal muscle hyposialylation.^8^^,^^14^ No renal phenotype has been associated with this genetic disorder, highlighting interspecies difference in SA biosynthetic pathways in podocytes.^1^^,^^8^ Clinical trials of ManNAc for GNE myopathy (NCT01634750, NCT02346461) showed long-term safety, increased plasma SA and sarcolemmal sialylation and improved muscle strength.^6^^,^^36^ However, PK data in individuals with reduced kidney function and reduced eGFRs are lacking because of normal kidney function in subjects with GNE myopathy.

Given ManNAc’s ability to enhance sialylation and its preclinical efficacy in nephrotic models, we launched a phase 1 trial (NCT02639260) to assess its safety, tolerability, and PKs in primary glomerular diseases across varying renal function.

## Methods

### Trial Design

**Figure 2:**
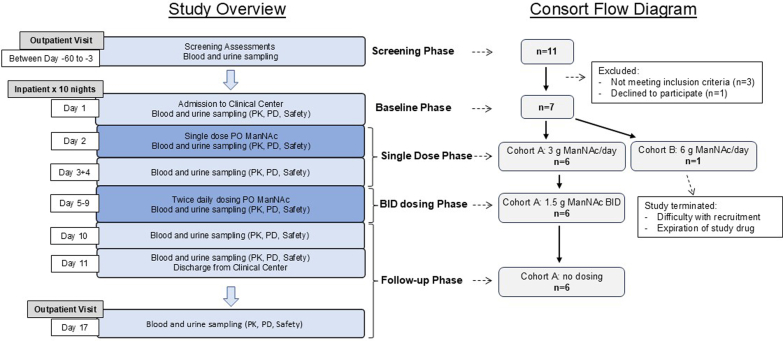
Clinical study overview and consort flow diagram. Subjects were screened for eligibility 3 to 60 days before dosing. After eligibility was confirmed and informed consent was obtained, subjects were admitted to the National Institutes of Health Clinical Center for an 11-day inpatient stay. Baseline assessments were performed on day 1, followed by administration of a single ManNAc dose (day 2), a wash-out period (days 3 and 4), BID oral ManNAc dosing for 5 days (days 5–9), an off-drug observation period (days 10–11), and discharge as an inpatient on day 11. Subjects returned to the National Institutes of Health Clinical Center for a 1-day outpatient visit on study day 17. Throughout the study period, safety monitoring was conducted via adverse events reporting, blood and urine laboratory tests, and clinical evaluation. PK was analyzed for plasma ManNAc and free sialic acid (Neu5Ac) at various time points; the effects of reduced eGFR on these plasma metabolites were assessed. BID, twice daily; ManNAc, N-acetylmannosamine; PD, pharmacodynamics; PK, pharmacokinetics; PO, by mouth.

This was a single-center, nonrandomized, single-arm, multiple ascending dose trial under National Institutes of Health protocol 16-DK-0036, “A Phase 1 Multiple Ascending Dose Study to Evaluate the Safety, Tolerability, and Pharmacokinetics of ManNAc in Subjects with Primary Podocyte Diseases” (ClinicalTrials.gov NCT02639260). The protocol was approved by the National Institute of Diabetes and Digestive and Kidney Diseases Institutional Review Board at the National Institutes of Health. The study was done in compliance with the International Council on Harmonization Good Clinical Practice guideline, and consistent with the Declaration of Helsinki. Written informed consent was obtained from each patient before enrolment. ManNAc was administered under US Food and Drug Administration Investigational New Drug (IND) 125,192. The study rationale and design were previously described^17^; in Figure 2, we present a study overview.

### Participants

Eligible participants were aged > 18 years, weighed > 40 kg, with biopsy-confirmed primary minimal change disease, FSGS (all variants including collapsing, tip, cellular, perihilar, and NOS, as well as adaptive or genetic forms), or membranous nephropathy. Additional criteria included UPCR > 1 g/g and eGFR ≥ 15 ml/min per 1.73 m^2^ (2012 Chronic Kidney Disease (CKD)-Epidemiology Collaboration Cr/CysC equation) (Table 1). Both steroid-sensitive and steroid-resistant individuals were eligible, those on steroids were asked to remain on a stable dose. Concomitant therapies, including immunosuppressants and renin-angiotensin system blockers, were permitted if stable from day −30 to +31.

Exclusion criteria were any patient with prior organ or stem-cell transplantation; requirement for IV diuretics; HIV, hepatitis B virus, or hepatitis C virus positivity; aspartate aminotransferase, alanine aminotransferase, or gamma-glutamyl transferase > 3 × ULN; pregnancy or breastfeeding; or use of other investigational drug or device, i.v.Ig, or SA-containing supplements, within 60 days before dosing. Full eligibility criteria are presented in Supplementary Table S1.

### Intervention

ManNAc (N-acetyl-D-mannosamine monohydrate; formerly DEX-M74; molecular weight 239.2 g/mol) was manufactured under GMP (New Zealand Pharmaceuticals, Palmerston North, New Zealand) and dispensed by the National Institutes of Health Clinical Center Pharmacy. Oral dosing involved reconstitution in 200 ml sterile water. All doses were administered during an 11-day inpatient stay (National Institutes of Health Clinical Center, Bethesda, MD). Study phases included screening, baseline, single-dose, multiple-dose, and follow-up, as outlined in Figure 2. In the single-dose phase (days 2–4), participants received 3 or 6 g ManNAc at 8:00 AM. In the multiple-dose phase (days 5–9), participants received 1.5 or 3 g ManNAc BID (8:00 AM fasting, 8:00 AM ≥ 2 h postprandial).

A safety review committee would evaluate the safety data from the first 2 patients after receiving a single dose (days 1–4) in each cohort and if deemed safe, would proceed to 5 days of BID dosing (days 5–11). If the safety data were favorable, the remaining cohort members would proceed directly to their full 11-day inpatient stay, including both the single-dose and BID-dose phases. Progression to cohort B (6 g ManNAc/d) would proceed only after the safety review committee had reviewed cohort A (3 g ManNAc/d) safety data through day 11.

### Sample Size

The sample size for this first-in-human phase 1 dose-escalation study was not determined by a formal power calculation, because the primary objective was to evaluate the safety, tolerability, and PKs of ManNAc in patients with varying degrees of renal function, rather than to test for a statistically significant effect. The trial followed a standard dose-escalation design, enrolling 6 patients in cohort A (3 g/d) and 6 in cohort B (6 g/d), with stratification by eGFR: 4 patients in each cohort with eGFR ≥ 30 ml/min per 1.73 m^2^ and 2 patients in each cohort with eGFR of 15–29 ml/min per 1.73 m^2^. This sample size was considered as ethically appropriate, keeping the number of participants to minimum to establish a maximum tolerated dose and define a recommended dose for future phase 2 trials. Preclinical data and PK modeling guided the starting dose and escalation steps, providing a robust rationale for the selected dose levels.

### Outcomes

**Figure 3:**
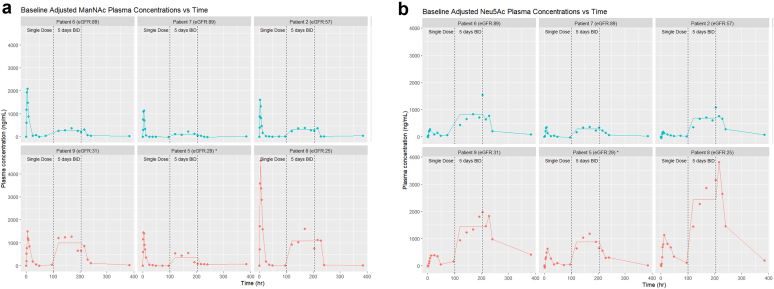
Pharmacokinetic profiles of plasma ManNAc and Neu5Ac. Plasma concentration-time profiles of (a) ManNAc and (b) Neu5Ac after oral administration of a single dose of 3 g oral ManNAc (*t* = 0 h), followed by 5 days of 1.5 g ManNAc twice-daily dosing (between *t* = 96–204 h), followed by a washout period of 1 week (*t* = 384 h). Graphs show absolute (nonbaseline-subtracted) values of each metabolite at each time point. Patients with eGFR > 45 ml/min per 1.73 m^2^ are displayed in the top rows (teal graphs) and subjects with eGFR < 45 ml/min per 1.73 m^2^ in the bottom rows (red graphs). For the 5-day twice-daily dosing (between dotted lines) each graph displays mean values, to help interpret steady state values of this dosing regimen. ∗Patient 5 stopped the twice-daily dosing phase after the morning dose of study day 8 (= day 4 of twice-daily dosing; *t* = 192 h). eGFR, estimated glomerular filtration rate; ManNAc, N-acetylmannosamine.

Primary outcomes included safety and tolerability of single or multiple oral ManNAc doses, maximum tolerated dose, and PKs. Safety and tolerability assessments included AEs, vital signs, physical exams, electrocardiograms, and blood and urine laboratory test results, with additional evaluation on day 17 or 1-week postdose. Drug escalation to the 6 g dose cohort required ≤ 1 moderate drug-related AE and no severe or serious AEs. All dosing was directly observed during the inpatient stay; subjects-maintained AE and compliance diaries, reviewed with case report forms. PK assessments involved ManNAc and free (unconjugated) SA (Neu5Ac) quantitation in plasma samples, performed by using liquid chromatography-mass spectrometry, using validated bioanalytical methods (Alliance Pharma).^37^ PK parameters (Table 2) were calculated using noncompartmental method with Phoenix WinNonLin, Version 6.2.0 (Certara, St. Louis, MO). Concentration-time graphics were generated in R v4.3.3. (Figure 3).^38^

Secondary or exploratory outcomes included pharmacodynamic effects on serum creatinine, eGFR, serum albumin, total cholesterol, triglycerides, hematocrit, uric acid, calcium, blood cell counts, and UPCR at baseline (days 1–2) and end of treatment (days 10–11).

Glomerular sialylation analysis was performed on an exploratory base on unstained, formalin-fixed, paraffin-embedded sections from a diagnostic kidney biopsy taken before this study, that were obtained for each subject. The sialylation status of glomeruli on the biopsy sections were semiquantified by histochemistry of fluorescein isothiocyanate–labeled lectin SNA (elderberry bark agglutinin from *Sambucus nigra*, binding terminal α[2,6]-linked SA[Neu5Ac] end groups on glycans)^39^ and the podocyte marker CD151 (a tetraspanin protein) as described in the Supplementary Methods.^7^^,^^17^ No kidney biopsies were obtained after ManNAc dosing in this phase 1 trial.

### Statistical Methods

Statistical analysis methods are described in the Supplementary Methods.

## Results

### Enrollment and Demographics

A participant flow diagram for this study is presented in Figure 2. All 6 patients planned for cohort A were recruited and completed the study (between July 2016 and November 2017). Safety data were analyzed before proceeding to cohort B. We could enroll only 1 patient in cohort B (December 2017), who completed only the single dose phase (days 1–4) of the study. Because no further patients volunteered and/or were found to be eligible for the study, before the expiration of our study drug (May 2018), we could not proceed any further on investigating this dose cohort, including BID phase for patient MAN10. The study was closed for enrollment in July 2018.

**Figure 4:**
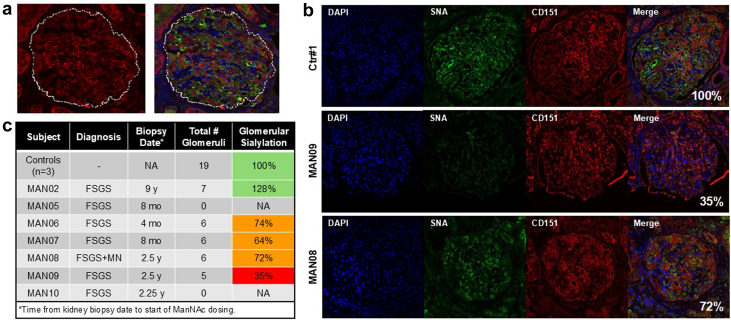
Lectin staining to quantify glomerular sialylation. Paraffin-embedded slides from a prior kidney biopsy of all 7 subjects and 3 controls were stained with fluorescein isothiocyanate–labeled SNA lectin (green: binding to terminal 2,6-linked sialic acid groups on glycans) and CD151 antibodies (red: a podocyte membrane marker) and the nuclear dye DAPI (blue; to indicate distribution of viable cells). Staining and imaging details are presented in the Supplementary Methods. (a) Example of a collapsed Z-stack of 7-slices of confocal images with a manual-drawn glomerular region of interest (ROI, white), used for fluorescence quantification in SNA/CD151 colocalizing areas, as described in the Supplementary Methods. Left image: single channel imaging of AlexaFluor 555 dye (red), representing CD151-positive areas. Right image: overlay image of all 3 fluorescent channels. (b) Representative confocal images of glomeruli from a control and 2 subjects’ biopsy slides, stained and imaged under the same conditions. Note the reduction of SNA intensity (green), but similar intensities of CD151 (red) and DAPI (blue) in the subjects’ images compared with control. (c) Summary of the 3 control and 7 patient kidney biopsy glomerular sialylation results, quantified and normalized to control biopsy slides. Green = normal sialylation; orange = moderate hyposialylation; red = severe hyposialylation. FSGS, focal segmental glomerulosclerosis; MN, membranous nephropathy. NA, not applicable.

Characteristics for all 7 participants are summarized in Table 1. Patients’ age range was 37 to 74 years (mean: 50.9 years) and included 5 males and 2 females. Self-reported ethnicity was African American in 3 subjects and non-Hispanic White in 4 subjects. Baseline eGFR values were 25 to 89 ml/min per 1.73 m^2^, and baseline UPCR ranged from 1.11 to 9.21 g/g. In cohort A, 2 patients had stage 4 CKD, 2 had stage 3 CKD, and 2 had stage 1/2 CKD; whereas the single cohort B patient had stage 3 CKD. Renal biopsy confirmed FSGS in 5 cohort A patients and FSGS with membranous nephropathy in 1; the only patient in cohort B had FSGS (Table 1). Time from biopsy to enrollment ranged from 4 to 108 months (median 29 months, Figure 4c). Participant’s concomitant medications are listed in Supplementary Table S3. Five out of the 7 treated subjects had received prior immunosuppression (steroids, mycophenolate mofetil, cyclosporine A, or rituximab) and renin-angiotensin-aldosterone blockade; none were on SGLT2 inhibitors.

**Table 1:** Participant characteristics

Subject^a^	Sex	Age^b^ (yr)	Weight^b^ (kg)	Diagnosis^c^	Glomerular sialylation^d^	ManNAc dose^e^	eGFR (ml/min per 1.73 m^2^)			UPCR (g/g)		
							BL	D10	D17	BL (mean D1+2)	End dosing (mean D10+11)	1 wk postdosing D17
Cohort A												
MAN06	F	47	87	FSGS	74%	3 g	89	78	70	2.72	1.30	1.05
MAN07	M	37	105	FSGS	64%	3 g	89	79	86	3.37	2.75	2.14
MAN02	M	57	71	FSGS	128%	3 g	57	59	51	3.27	3.91	3.33
MAN09	M	74	89	FSGS	35%	3 g	31	26	26	9.21	4.87	3.47
MAN05	M	45	135	FSGS	NA	3 g	29	24	26	2.66	1.79	2.66
MAN08	M	64	138	FSGS + MN^f^	72%	3 g	25	27	24	6.04	5.31	6.23
Cohort B												
MAN10	F	42	144	FSGS	NA	6 g	56	NA	NA	1.11	NA	NA

BL, baseline; D, study day; eGFR, estimated glomerular filtration rate; F, female; FSGS, focal segmental glomerulosclerosis; M, male; ManNAc, N-acetylmannosamine; MN, membranous nephropathy; NA, not applicable; NIH, National Institutes of Health; UPCR, urine protein-to-creatinine ratio.

a Noncontiguous numbering of subjects is due to some enrolled subjects failing inclusion or exclusion criteria in the prescreening or screening stages of the study.

b On day 1 predose.

c Based on pathology reports of a diagnostic prestudy biopsy. For subject MAN02, the pathology report was missing; the diagnosis was made by NIH pathology on prestudy biopsy slides. All patients demonstrated nephrotic-range proteinuria and hypoalbuminemia at the time of biopsy. All biopsy reports indicated diffuse foot process effacement, and none indicated features indicative of adaptive FSGS.

d Kidney slides from a biopsy prior to protocol enrollment was assessed for glomerular sialylation status (Figure 4).

e All subjects in cohort A received a single dose of 3 g ManNAc (day 2), followed by 1.5 g ManNAc twice daily (days 5–9). Subject MAN10, in cohort B, only received a single dose of 6 g ManNAc.

f Subject, MAN08 was diagnosed with primary MN in conjunction with FSGS. The FSGS in this subject may represent a secondary manifestation of MN.

**Table 2:** Plasma PK parameters of a single 3 g ManNAc dose in subjects with primary podocyte disease and decreased eGFR (< 90 ml/min per 1.73 m^2^; current study) compared with values from previous studies of subjects with normal eGFR (12-HG-0207 and 15-HG-0068)

			Plasma ManNAc				Plasma Neu5Ac (sialic acid)			
Study	eGFR	ManNAc dose	T_max_^a^ (h)	C_max_^b^ (ng/ml)	AUC_last_^c^ (ng∗h/ml)	t_1/2_^a^ (h)	T_max_^a^ (h)	C_max_^b^ (ng/ml)	AUC_last_^c^ (ng∗h/ml)	t_1/2_^a^ (h)
12-HG-0207^d^	> 90	3 g (*n* = 4)	2.0	1451	8244	2.2	10	115	2147	5.5
15-HG-0068^e^	> 90	3 g (*n* = 6)	2.0	1356	7438	3.2	6.0	258	2506	14
Current study	30–90	3 g (*n* = 4)	3.5	1579	14,835	4.0	11.9	301	7474	f
	15–29	3 g (*n* = 2)	3.0	3020	30,248	6.3	12.0	876	26,826	14

eGFR, estimated glomerular filtration rate; ManNAc, N-acetylmannosamine; PK, pharmacokinetics.

PK calculations were performed with baseline adjustment by subtracting ManNAc or Neu5Ac concentrations at *t* = 0 for each subject.

a Median.

b Geometric mean.

c Mean.

d Ref.^6^

e Ref.^36^

f Unable to calculate: no minimum of 3 descending Neu5Ac concentration time points for MAN07 and MAN09.

### Safety and Tolerability

Oral ManNAc was well-tolerated without any serious or severe AEs occurring. No clinically relevant changes in electrocardiograms or vital signs were noted, including orthostatic heart rate or blood pressure (Supplementary Table S2).

All AEs observed in the study were either Common Terminology Criteria for Adverse Events Grade 1 (mild; asymptomatic or mild symptoms) or Grade 2 (moderate; minimal, local or noninvasive intervention indicated) (Table 3). No participants dropped out of the study. Mild AEs occurred in all study subjects, with only 2 subjects experiencing a moderate AE. Headache and gastrointestinal side effects, including diarrhea, loose stools, nausea, and bloating, were common, as was found in previous studies investigating ManNAc for use in GNE myopathy.^6^^,^^36^ One subject (MAN05) in the low eGFR category (eGFR: 15–29 ml/min per 1.73 m^2^) had drug administration terminated early because of excessive fatigue (Table 3).

**Table 3:** Adverse events by subject, relation to study drug and CTCAE grade

Subject number	Adverse event	Relatedness to study drug^a^	CTCAE Grade^b^	Details and outcome
MAN02	Nausea	Possible	1	Resolved within 3–6h
	Myalgia	Possible	1	Cramp resolved with position change
	Headache	Possible	1	Resolved with 650 mg acetaminophen
	Pain in an extremity	Possible	1	Resolved without intervention
	Rash maculopapular	Possible	1	Rash on right forearm. Resolved without intervention
MAN05^c^	Pain in extremity	Possible	1	Gout-related pain in toe. Resolved; Uric acid within normal limits
	Glucose intolerance	Unlikely	1	Resolving: does not meet threshold for diabetes
	Nausea	Possible	1	Resolved: relieved by single dose of antacid and sleep
	Fatigue	Probable	1	Resolved 26 h after final study drug dose (on day 8; subject was halted taking further ManNAc)
	Platelet count decreased	Possible	1	Resolved: platelet count within normal range on day 17
	Tremor	Possible	1	Resolved: decreased significantly within 2 d off ManNAc. Subject was halted taking further ManNAc (on day 8)
	Anal hemorrhage	Possible	1	An episode of small amount of blood from rectum noted when wiping anus, no additional bleeding noted
	Dizziness	Possible	1-2	Dizziness Grade 2 began ∼2 h after discharge from NIH Clinical Center (day 11). Grade 1 on study days 12–14. Resolved without intervention by study day 15
	Generalized muscle weakness	Possible	1	Subject reported weakness ∼2 h after discharge from NIH Clinical Center (day 11), resolved by next day
	Gastroesophageal reflux	Possible	1	Heartburn reported on days 12 and 13 that resolved without intervention by day 14
	Urinary Tract pain	Possible	1	A stinging sensation during urination was reported on day 13 that resolved without intervention by day 15
MAN06	Diarrhea	Probable	1	Single episode on day 2, 2 h after 3 g ManNAc dose. Resolved: Without intervention, bowel pattern returned to normal after 5 h
MAN07	Flatulence	Possible	1	Resolved: subject reported occasional flatulence during hospitalization
	Fatigue	Possible	1	Resolved: fatigue relieved by rest on study days 2 and 5, each episode lasted about 4 h
	Bloating	Possible	1	Resolved: bloating lasting about 4 h
	Constipation	Possible	1	Resolved: constipation lasting about 2h
	Headache	Possible	1	Resolved: subject reported 2 episodes of headache lasting about 1 h each, resolved without treatment
MAN08	Hypercalcemia	Possible	1	Resolved: subject’s home medication calcium dose was different from NIH pharmacy dose. Discovered after rise in serum calcium was noted. Calcium supplement was held until discharge; home medications were restarted. Total and ionized calcium were in normal range at the day 17 follow-up visit
MAN09	Hypertension prior to study drug	NA	2	Hypertension with SBP > 140 mm Hg. Resolved after medication change
	Bradycardia prior to study drug	NA	1	Cardiology consultation, beta-blocker drug changed. Resolved after medication change
	Cough	Possible	1	Intermittent cough throughout inpatient stay, no intervention necessary. Resolved
	Dry mouth	Possible	1	Resolved
	Headache	Possible	1	Headaches off and on throughout inpatient stay Acetaminophen given as needed for headache
	Fever	Possible	1	Resolved. Acetaminophen given; fever did not return
	Lung infection prior to study drug	NA	1	Sputum culture collected on admission, positive for *Pseudomonas* (patient has history of colonization with this organism) and *Escherichia coli*. No intervention provided
	Investigations - Other, CRP increased	Possible	1 and 2	CRP elevated on day 4, increased to > 95 on days 5–9, decreased to 39.4 on day 11. ESR was also elevated. Blood and urine cultures were negative, and chest x-ray did not show evidence of pneumonia. On day 17, CRP was down to 2.80 (normal range)
	Investigations - Other, alkaline phosphatase increased	Possible	1	Alkaline phosphatase increased on study days 7 and 8 (139–141) returned to normal on day 9
	Rash	Possible	1	Small red macule on upper chest beginning on day 3, improving by day 11. Rash had resolved on study day 17 outpatient follow-up
	Dyspnea	Possible	1	Mild dyspnea on exertion from days 7–11, subject attributed this to lack of activity. Dyspnea reported until study day 31, intermittently
MAN10	Loose stool	Possible	1	Patient reports 2 episodes of loose stools on day 4 (nondosing day). Symptoms resolved the same day

CRP, C-reactive protein; CTCAE, Common Terminology Criteria for Adverse Events; ESR, erythrocyte sedimentation rate; ManNAc, N-acetylmannosamine NIH, National Institutes of Health; SBP, systolic blood pressure.

a Relatedness to study drug values: None; Unlikely; Possible; Probable; Definite.

b CTCAE values: 1 = Mild; 2 = Moderate; 3 = Severe; 4 = Potentially life-threatening; 5 = Death.

c Subject MAN05, in the low eGFR category (eGFR: 15–29 ml/min per 1.73 m^2^) had drug administration terminated early because of excessive fatigue. This was first noted about 8 h after the administration of the first dose on day 5 and continued for the next 72 h, at which time it was decided to hold further doses. This resolved about 26 h after the administration of the last dose. The subject was discharged on day 11 as scheduled per protocol.

### PKs

The PK profiles of plasma ManNAc and Neu5Ac (SA) are displayed in Figure 3 and PK parameters are shown in Table 2. Baseline plasma ManNAc levels in patients with eGFR > 45 ml/min per 1.73 m^2^ (Figure 3a, top panel) were similar to published values for individuals with GNE myopathy, who had normal renal function (eGFR > 90 ml/min per 1.73 m^2^).^6^ After a single dose of 3 g ManNAc, plasma ManNAc levels peaked in most subjects 2 to 4 hours after dosing and returned to baseline after approximately 12 hours. Plasma ManNAc did not significantly accumulate following BID dosing in this group of subjects. However, in subjects with eGFR < 45 ml/min per 1.73 m^2^, ManNAc levels reached higher maximum plasma concentrations, resulting in a higher drug exposure (AUC_last_) and a longer plasma half-life of ManNAc (*t*_1/2_) than in subjects with higher eGFR values (Table 2, Figure 3a bottom panel).^6^

Baseline plasma Neu5Ac levels were elevated in subjects with reduced eGFR. This is consistent with the predominantly glomerular clearance of Neu5Ac with minimal tubular reabsorption (like creatinine), an often-overlooked aspect of Neu5Ac renal filtering.^40^ Plasma-free Neu5Ac levels peaked 8 to 12 hours after dosing, remained elevated beyond 48 hours, and continued to increase during BID dosing, particularly in subjects with eGFR < 45 ml/min per 1.73 m^2^ (Figure 3B). Subjects with eGFR < 45 ml/min per 1.73 m^2^ had significantly increased maximum plasma ManNAc concentration, AUC_last_, and t_1/2_ values compared with subjects with higher eGFRs (Table 2); plasma-free Neu5Ac levels reached markedly high levels, although there appeared to be no AEs associated with these increased plasma levels during the approximately 5 days of exposure.

Mean plasma concentrations during the 5-day BID dosing phase are shown in Figure 3a and b. Both ManNAc and Neu5Ac levels reached a steady state in subjects with eGFR > 45 ml/min per 1.73 m^2^ during this time period. Neu5Ac levels in subjects with eGFR > 45 ml/min per 1.73 m^2^ (Figure 3b, upper panel) reached elevated steady state trough levels < 1500 ng/ml during the BID dosing period and returned to baseline after the 1-week washout period. However, Neu5Ac trough levels in subjects with eGFR < 45 ml/min per 1.73 m^2^ continue to rise during the BID dosing phase (Figure 3b, bottom panel) and reached significantly higher levels (up to > 4000 ng/ml), continuing to accumulate after 5 days of BID dosing, and in some subjects not returning to baseline after the 1-week washout period.

### Glomerular Sialylation Analysis

Glomerular sialylation was assessed in slides from kidney biopsies obtained prior to protocol enrollment. In all glomeruli present on each slide, the fluorescence intensity of SNA lectin (2,6-linked SA groups bound to glomerular glycans) was quantified on podocyte membranes as identified by CD151 positivity (Supplementary Methods and Figure 4a and b).

One patient (MAN09) had severely hyposialylated glomeruli (35% residual SNA signal compared with controls), 3 patients (MAN06, MAN07, and MAN08) showed moderate hyposialylation (74%, 64%, 72% of control, respectively), 1 patient (MAN02) had normal sialylation, and the biopsy slides of 2 patients (MAN05 and MAN10) contained no glomeruli and were not informative for this analysis (Figure 4c).

### Pharmacodynamics

Relevant changes in the clinical laboratory blood and urine test results between baseline and end-of-dosing were assessed on an exploratory basis in this limited group of subjects. For most test results, there were no clinically relevant changes, including creatinine, eGFR, serum albumin, total cholesterol, triglycerides, hematocrit, uric acid, calcium, and blood cell counts (Supplementary Table S2).

**Figure 5:**
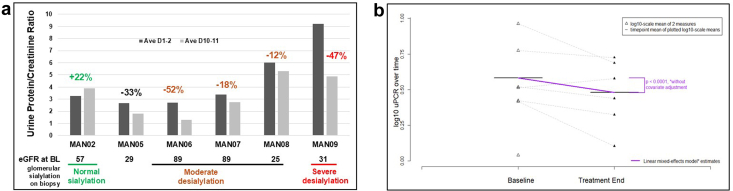
Changes in UPCR over the trial duration. (a) UPCR values at baseline (dark gray; average of spot-urines on predose days 1 and 2) and at the end of treatment (light gray; average of first morning voids on days 10 and 11) on a raw scale showing that most subjects (5 of 6) who received ManNAc twice daily had a marked reduction in UPCR (g/g) (12%–52%). Note that all subjects who had moderate or severe hyposialylated glomeruli on a prestudy kidney biopsy (Figure 4) showed a reduction in proteinuria; the one subject (MAN02) with normal sialylated glomeruli did not exhibit reduced proteinuria in this study. (b) Plot of changes in UPCR between baseline (mean of days 1 and 2) to end of treatment (mean of days 10 and 11) on log_10_-transformed scale across 6 recipients that received ManNAc twice daily; model-estimated mean decrease in UPCR amounted to 9.7% (95% confidence interval: 5.5%–13.65%, *P* < 0.0001. ∗Analyzed via mixed-effects repeated measures linear regression model of log_10_ UPCR, allowing for between-participant heterogeneity via random intercepts (Supplementary Table S4). BL, baseline; D, day; eGFR, estimated glomerular filtration rate; UPCR, urine protein-to-creatinine ratio.

However, there was a trend toward reduced proteinuria in most subjects receiving 1.5 g ManNAc BID for 5 days, as demonstrated by a change from baseline (days 1–2) to end of treatment (days 10–11) in UPCR (Figure 5, Supplementary Figure S1), urine albumin, and urine albumin-to-creatinine ratio (Supplementary Table S2). There was a trend toward reduction in UPCR at the end of therapy in a majority of cohort A patients receiving the BID dosing, with 5 of 6 showing a reduction of 12% to 52% on raw scale as compared individual baseline values (Figure 5a). The model-estimated mean reduction in UPCR across all 6 subjects was 9.7% with a 95% confidence interval: 5.5%–13.7%, *P* < 0.0001) (Figure 5b, Supplementary Table S4, Supplementary Statistical Methods). This reduction was analyzed using all available values and a mixed-effects repeated measures linear regression model of UPCR values on the log_10_-transformed scale, adjusting for screening values of log_10_UPCR as well as baseline values of eGFR and plasma Neu5Ac. Between-participant heterogeneity was accommodated by employing random intercepts, using PROC MIXED in SAS v9.4 (SAS Institute Inc, Cary, NC) and corroborated using lme4 in R v.4.5.1. (R Core Team, 2024).^38^

The degree of proteinuria reduction correlated with the degree of baseline glomerular hyposialylation (exact linear-by-linear association test at the 5% significance level, implemented in StatXact v8 (Cytel Inc. Cambridge, MA), corroborated in SAS v9.4 by a glomeruli-count-informed exact Jonckheere-Terpstra test,^41^ significant at the 0.5% level; Supplementary Statistical Methods). The 4 subjects with reduced glomerular sialylation (MAN06, MAN07, MAN08, and MAN09) had a reduction in UPCR after receiving ManNAc BID, whereas the one subject (MAN02) with normal sialylation did not show a UPCR reduction (Figures 4 and 5a).

## Discussion

Podocyte injury plays a pivotal role in primary and secondary proteinuric kidney diseases. Current management of primary podocytopathies such as FSGS largely relies on repurposed immunosuppressants, reflecting an assumed immunologic pathogenesis. However, emerging mechanistic insights highlight the structural role of the podocyte slit diaphragm, where the negatively charged glycocalyx is a major contributor to charge-selective filtration.^4^ This dense glycocalyx is enriched with SA residues on the terminal ends of key podocyte glycoproteins such as podocalyxin and nephrin. In animal models, podocyte desialylation led to massive proteinuria and FSGS-like histology, due to reduction in podocyte surface negative charge as well as impaired cell proliferation, differentiation, and/or collagen type IV adhesion.^42^ Oxidative stress has been suggested as causative for glycan desialylation,^5^^,^^14^^,^^43^ including glycans in the glomerular glycocalyx.^20^, ^21^, ^22^^,^^44^^,^^45^ In the aminonucleoside-induced nephrosis model it was shown that exogenous SA supplementation not only led to resialylation of podocyte glycocalyx, but also rebalanced the oxidative milieu by upregulation of endogenous mSOD.^45^

The development of ManNAc therapy for primary podocyte diseases began with preclinical evidence that SA depletion gave rise to proteinuria in nephrotic animal models.^7^^,^^8^^,^^27^, ^28^^,^^30^^,^^31^^,^^46^, ^47^, ^48^ and that oral administration of ManNAc restored sialylation and reduced proteinuria in these models.^7^, ^8^, ^9^^,^^29^^,^^32^^,^^34^^,^^35^ Given that altered podocyte sialylation in human glomerulopathies was already demonstrated in past^15^^,^^18^ and in recent studies,^17^^,^^19^, ^20^, ^21^, ^22^, ^23^ we considered exploring ManNAc to augment intrapodocyte SA synthesis as a novel targeted therapy to restore podocyte structure and function, that could translate into improved clinical outcomes.^17^ Human safety data on ManNAc therapy are available extending over a period of 30 months, emanating from clinical trials in GNE myopathy, where no serious adverse effects were noted (IND 78,091).^6^^,^^36^

Encouraged by the preclinical data and human safety profiles of ManNAc, we initiated this investigator-sponsored phase 1 trial (IND 125,192) to assess safety, tolerability, and PK of ManNAc in subjects with primary podocytopathies. The study primarily aimed to explore this novel agent in the space of glomerular disease in a small but diverse ethnic mix of subjects, with proteinuria ranging from subnephrotic to overtly nephrotic (1.3–9.9 g/g) and with eGFR from 25 to 89 ml/min per 1.73 m^2^, allowing assessment of these variables on drug metabolism, elimination, and safety. Data were obtained predominantly for the 3 g daily dose group (*n* = 6) because the sole participant in the 6 g arm was withdrawn after a single dose. Oral ManNAc 1.5 g BID proved to be generally safe with mild gastrointestinal side effects in nephrotic patients, similar to previous studies of ManNAc use in patients with GNE myopathy.^36^ Only 1 patient discontinued ManNAc because of investigator concerns about excessive fatigue.

SA clearance occurs primarily by glomerular filtration, unlike other monosaccharides such as glucose, mannose, galactose, and fructose that exhibit substantial tubular reabsorption.^40^^,^^48^ Likely because of this phenomenon, SA continued to accumulate and did not reach steady state within the 5-day BID drug administration in subjects with low eGFRs (< 45 ml/min per 1.73 m^2^) (Figure 3), necessitating future longer-term PK studies. Plasma-free SA levels of approximately 100 to 200 ng/ml are considered normal for eGFR > 90 ml/min per 1.73 m^2^,^37^^,^^40^ but we do not know what levels are deleterious. In SA storage disorders because of defects in the lysosomal-free SA transporter SLC17A5, plasma-free SA levels are chronically elevated in the range of 618 to 3059 ng/ml (*n* = 8).^48^ The disease phenotype includes neurodevelopmental delay, ataxia, epileptic seizures, and hepatosplenomegaly^49^; however, these manifestations may be related to storage of SA within lysosomes of specific cell types (such as central nervous system neurons), rather than to elevated circulating free SA levels.

In the present study, there was no correlation between the frequency or severity of AEs and the increase in plasma-free SA levels. In addition, patients with GNE myopathy (without nephropathy and with eGFRs > 90 ml/min per 1.73 m^2^) who received 6 g ManNAc BID for 30 months had moderately elevated plasma SA levels (∼250–900 ng/ml), but did not experience serious adverse clinical or biochemical events.^36^ However, plasma SA levels are likely to be higher in individuals with eGFR values < 45 ml/min per 1.73 m^2^, both in the treated and untreated states, so caution is warranted when using ManNAc in this group (Figure 3).^40^ Based on the PK data, we recommend that future studies use ManNAc at < 6 g/d in subjects with eGFR between 45 and 90 ml/min per 1.73 m^2^, with close monitoring of subjects with lower eGFRs.

Although this study aimed to generate preliminary drug-safety data for ManNAc in patients with a primary glomerular disease, trends of clinical efficacy were also noted. Proteinuria reduction (12-52%) was observed in 5 of the 6 subjects given 1.5 g ManNAc BID over 5 days (Figure 5a). A limitation was the use of spot UPCR for proteinuria assessment, which may show only modest correlation with gold-standard 24-hour estimates.^50^ To address this limitation, we employed models that account for between-individual variability alongside other predictive measures, thus used the following: (i) baseline UPCR as the mean of time point–adjacent values for spot-urines on day 1 and day 2, and (ii) for at end-of-ManNAc exposure UPCR, we used the mean of time point–adjacent values for first morning voids at day 10 and day 11 (Figure 5a, Table 1). In addition, using the log_10_-transformed scale of UPCR values, which have a stronger association with 24-hour protein excretion,^50^^,^^51^ we estimated that proteinuria mean levels across these time points decreased by approximately 9.7% (95% confidence interval: 5.5%–13.7%, *P* < 0.0001 (Supplementary Table S4), based on a linear mixed-effects repeated measures model leveraging all available log-transformed UPCR (key time points–based estimates depicted in Figure 5b). Moreover, the degree of proteinuria reduction appeared to correlate with the extend of glomerular hyposialylation in pre-treatment biopsies (Figures 4 and 5a, Supplementary Figure S1; *P:* 0.005, Supplementary Statistical Methods) Note that the interval between biopsy and study enrollment was variable (median 29 months, Figure 4c), which may influence the interpretation of these exploratory findings. Nonetheless, the data suggest that baseline glomerular hyposialylation could help identify patients most likely to benefit from ManNAc therapy,^17^ analogous to the use of inaxaplin in APOL1-associated FSGS.^52^ These preliminary observations warrant confirmation in future studies.

Limitations of this study include the small participant sample size (thus, we used models leveraging all available PK or efficacy repeated measurements), absence of a control arm, absence of safety data on higher treatment doses (which could emerge as an issue in efficacy-trials), short treatment duration, and absence of posttreatment kidney biopsies. Because this trial was conducted before the widespread adoption of the race-free CKD-Epidemiology Collaboration 2021 equations, limitations in generalizability to current clinical practice are expected, particularly regarding correlations of PK data with eGFR. Considering these limitations, and given the reassuring safety data, a phase 2 study has been initiated to evaluate long-term safety, PK, and consistent efficacy signals in patients with primary FSGS (ClinicalTrials.gov NCT06664814), which will determine the feasibility of a subsequent randomized trial to confirm the efficacy of this novel agent.

## Conclusion

In conclusion, this phase 1 study provides evidence to confirm the short-term safety of ManNAc therapy in primary glomerular diseases while demonstrating a clear association of PK parameters with baseline eGFR. Early efficacy signals were observed in this limited cohort of patients that align with findings from animal models of glomerular hyposialylation, supporting further translational studies in proteinuric kidney diseases where strong mechanistic role has been shown such as primary podocytopathies,^17^^,^^19^^,^^32^^,^^34^ IgA nephropathy^17^^,^^24^^,^^25^ and diabetic kidney disease.^35^^,^^47^ In pursuit of safe, less toxic therapies, renal biopsy could emerge as a potential biomarker to identify patients with significant glomerular hyposialylation, enabling targeted, low-cost therapies to help reduce global burden and disparities in kidney diseases.^53^

## Appendix

### List of the NIH-ManNAc Study Team

Andrea Ashton, Jodi L Blake, Thomas A Cassini, and Kevin J O’Brien.

## Disclosure

MH, JBK, and WAG obtained funding through a Cooperative Research and Development Agreement with Escala Therapeutics. MH and WAG are coinventors on US patent 8,410,063, entitled “N-acetylmannosamine as a therapeutic agent,” with royalties paid to the National Human Genome Research Institute. All the other authors declared no competing interests.

## Supplementary Material

Supplementary File (PDF) **Supplementary Methods.** statistical analysis. **Supplementary Methods.** glomerular sialylation analysis. **Figure S1.** Changes in UPCR over the trial duration. **Table S1.** Full inclusion/exclusion criteria. **Table S2.** Clinical laboratory results - vital signs - ManNAc PK - Neu5Ac PK. **Table S3.** Participant concomitant medications. **Table S4.** Mixed effects repeated measures model estimates. CONSORT checklist.

## Footnotes

Supplementary File (PDF)
**Supplementary Methods.** statistical analysis. **Supplementary Methods.** glomerular sialylation analysis. **Figure S1.** Changes in UPCR over the trial duration. **Table S1.** Full inclusion/exclusion criteria. **Table S2.** Clinical laboratory results - vital signs - ManNAc PK - Neu5Ac PK. **Table S3.** Participant concomitant medications. **Table S4.** Mixed effects repeated measures model estimates. CONSORT checklist.
