# Supplementary material for: Phase 1 Study of Oral N-Acetylmannosamine in Primary Podocytopathies
Source: Kidney Int Rep. 2025 Dec 31;11(3):103758. doi: 10.1016/j.ekir.2025.103758 (PMC12861195; doi:10.1016/j.ekir.2025.103758)
Supplement: Supplementary File (PDF) — Supplementary Methods. statistical analysis. Supplementary Methods. glomerular sialylation analysis. Figure S1. Changes in UPCR over the trial duration. Table S1. Full inclusion/exclusion criteria. Table S2. Clinical laboratory results - vital signs - ManNAc PK - Neu5Ac PK. Table S3. Participant concomitant medications. Table S4. Mixed effects repeated measures model estimates. CONSORT checklist. [file mmc1.pdf]

**SUPPLEMENTARY DATA**

**Figure S1:** Changes in UPCR over the Trial Duration.

**Table S1:** Full Inclusion/Exclusion Criteria.

**Table S2:** Clinical Labs - Vital Signs - ManNAc PK - Neu5Ac PK.

**Table S3:** Participant Concomitant Medications.

**Table S4:** Mixed Effects Repeated Measures Model Estimates.

**Supplementary Methods:** Statistical Analysis.

**Supplementary Methods:** Glomerular Sialylation Analysis.

**CONSORT checklist**.

**Figure S1:**

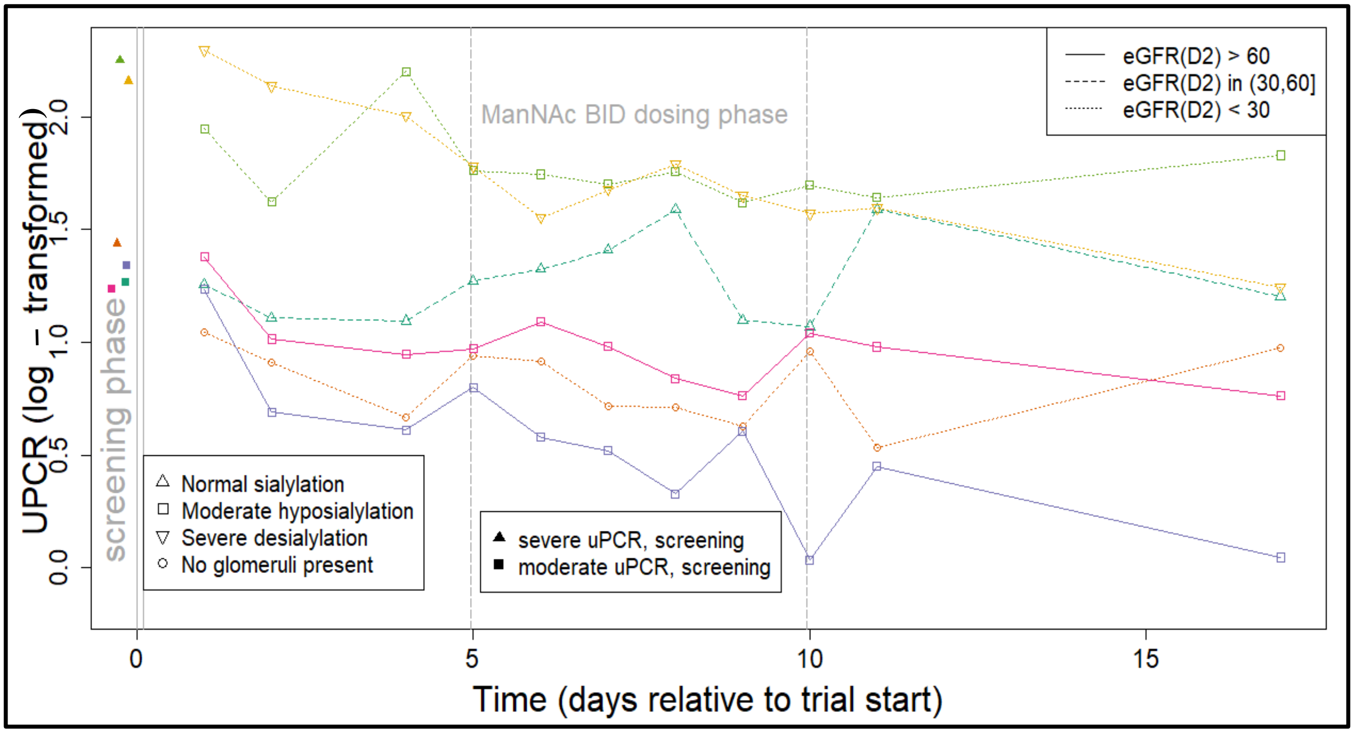


**Figure S1: Changes in UPCR over the Trial Duration**

Plot of log-transformed UPCR for each treated individual across time (*colored lines*), annotated with multiple variables of interest, including study epochs (*vertical lines*); severity of screening-phase proteinuria (*solid symbols*); baseline eGFR levels (*line type*); degree of biopsy hyposialylation (*hollow symbols*). Notably, the overall trends in proteinuria reflect decreases on average relative to baseline (plot generated using R v4.4.0).

# Table S1: Full Inclusion/Exclusion Criteria

## *Inclusion Criteria*

1. Prior kidney biopsy manifesting MCD, FSGS (including collapsing glomerulopathy) or MN is a requirement for inclusion. Kidney biopsy materials will be reviewed by NIH pathology to confirm the diagnosis. With regard to FSGS, all histologic variants, including collapsing, tip, cellular, perihilar and NOS will be included; primary, adaptive, and genetic FSGS will be included; viral-associated and drug-associated FSGS will be excluded. The rationale for including multiple primary nephrotic diseases is that the pharmacokinetics is likely to be similar and all these diseases have a need for effective therapy with low toxicity profile.
2. Age >18 years weighing more than 40 kg of either sex. The rationale for excluding children is that we are first seeking safety data for subjects with nephrotic and/or reduced GFR and evidence for benefit in proteinuric subjects.
3. Subjects must either not be taking immunosuppressive therapy (e.g., prednisone, cyclosporine, tacrolimus or mycophenolate mofetil) or, alternatively, be able to tolerate a stable dose of such a therapy from day -30 to day +31. If medically necessary, immunosuppressive therapy will be adjusted during the study. Subjects who are on renin angiotensin pathway inhibitor therapy will not be asked to discontinue their current regiment and will be included in the study.
4. Weight >40 kg. Subjects 40-60 kg will only be placed in the low-dose Cohort A. Subjects >60 kg can be placed in either Cohort A (3,000 mg/day) or Cohort B (10,000 mg/day). The rationale is to adhere to the maximum allowable starting dose of 193.5 mg ManNAc/kg body weight/day, derived from preclinical animal toxicology studies.
5. Subjects with first void urine protein/creatinine ratio > 1 g/g.
6. Subjects with an estimated glomerular filtration rate (eGFR) ≥15 mL/min/1.73 $m^2$ will be included. The rationale is that we wish to determine the effect of eGFR on ManNAc and Neu5Ac (sialic acid) metabolism (including plasma PK). We will compare 3 eGFR groups: subjects with stage 4 CKD (eGFR 15-29 mL/min/1.73$m^2$), stage 3 CKD (eGFR 30-59 mL/min/1.73$m^2$) and individuals with stage 1 or 2 CKD (eGFR ≥60 mL/min/1.73$m^2$). Therapy for individuals with stage 4 CKD is a particularly compelling unmet need, as many therapies become problematic (e.g., calcineurin inhibitors will further lower GFR and intensification of renin-angiotensin-aldosterone system inhibitors may lower GFR and raise serum potassium levels). eGFR will be assessed using serum creatinine (Cr) and cystatin C (CystC) using the CKD-EPI Cr/CystC equation for adults [Inker et al. Am J Kidney Dis. 2011;58(4):682-684].
7. Subjects must be able to comply with requirements of the protocol, including blood collection, drug administration, and effective communication with study staff.
8. Heterosexual couples must use at least one effective form of birth control, unless a hysterectomy, tubal ligation, or vasectomy has been performed. These may include the following: barrier methods, oral or an injection (for example, Norplant or Depo-Provera) contraception medication, and intrauterine devices.

## *Exclusion Criteria*

1. Unwilling or unable to provide informed consent.
2. Subject who requires use of intravenous diuretics to control edema, as this may result in fluid shifts between the intravascular space and the remainder of extracellular fluid volume. Oral diuretics will not

be exclusionary, and we reserve the option to use intravenous diuretics during the study if this becomes necessary.

3. Subject has a psychiatric illness or neurological disease that would interfere with the ability to comply with the requirements of this protocol. This includes, but is not limited to, uncontrolled/untreated psychotic depression, bipolar disorder, schizophrenia, substance abuse or dependence, antisocial personality disorder, panic disorder, or behavioral problems, which interfere with effective communication.
4. Vulnerable subjects, including those with impaired cognitive function or are incarcerated will be excluded.
5. Compromised venous access, such that it would interfere with peripheral intravenous access suitable for taking blood samples.
6. Subject has a severe disease manifestation that would interfere with the ability to comply with the requirements of this protocol.
7. Subjects with a positive HIV test including antibody or viral load.
8. Individuals whose blood contains HBV surface antigen or HCV antibody.
9. Subject has hepatic laboratory parameters (AST, ALT, GGTP) greater than 3 times the upper limit of normal.
10. Subject is anemic with hematocrit ≤30% (for both men and women).
11. Subject shows evidence of clinically significant cardiovascular, pulmonary, hepatic, renal, hematological, metabolic (including diabetes mellitus), or gastrointestinal disease, or has a condition that requires immediate surgical intervention.
12. Subject is pregnant or breastfeeding at any time during the study.
13. Subject has received treatment with another investigational drug, investigational device, or approved therapy for investigational use less than 60 days prior to ManNAc dosing.
14. Subject has a hypersensitivity to ManNAc or in the judgment of the investigator, has a condition that places the subject at increased risk for adverse events.
15. Subject has been treated with ManNAc, sialic acid, intravenous immunoglobulin (IVIG), and/or other supplements containing sialic acid (e.g., sialyllactose) less than 60 days prior to planned ManNAc dosing.

# Table S2: Clinical Labs - Vital Signs - ManNAc PK - Neu5Ac PK

## CLINICAL LABS

| | | no dosing | t=0 single dose | no dosing | no dosing | BID Day 1 | BID day 2 | BID day 3 | BID Day 4 | BID Day 5 | no dosing | no dosing | no dosing |
|---|---|---|---|---|---|---|---|---|---|---|---|---|---|
| **Urine protein** | **Screening** | **Day 1** | **Day 2** | **Day 3**** | **Day 4** | **Day 5** | **Day 6** | **Day 7** | **Day 8** | **Day 9** | **Day 10** | **Day 11** | **Day 17** |
| MAN02 | 117 | 165 | 109 | 178 | 215 | 157 | 158 | 86 | 142 | 159 | 140 | 211 | 160 |
| MAN05* | 509 | 310 | 176 | | 175 | 100 | 197 | 160 | 191 | 197 | 327 | 255 | 85 |
| MAN06 | 673 | 767 | 255 | | 92 | 249 | 198 | 230 | 258 | 176 | 88 | 224 | 136 |
| MAN07 | 358 | 151 | 651 | | 214 | 251 | 253 | 283 | 213 | 225 | 252 | 253 | 225 |
| MAN08 | 673 | 694 | 319 | | 334 | 256 | 275 | 225 | 226 | 283 | 191 | 222 | 455 |
| MAN09 | 381 | 507 | 280 | | 193 | 178 | 137 | 166 | 150 | 151 | 130 | 138 | 250 |
| MAN10 | | 44 | 56 | 65 | 96 | | | | | | | | |

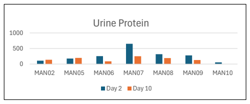


| Urine Creatinine | Screening | Day 1 | Day 2 | Day 3** | Day 4 | Day 5 | Day 6 | Day 7 | Day 8 | Day 9 | Day 10 | Day 11 | Day 17 |
|---|---|---|---|---|---|---|---|---|---|---|---|---|---|
| MAN02 | 33 | 47 | 36 | 43 | 72 | 44 | 42 | 21 | 29 | 53 | 48 | 43 | 48 |
| MAN05* | 121 | 109 | 71 | | 90 | 39 | 79 | 78 | 94 | 105 | 125 | 150 | 32 |
| MAN06 | 176 | 223 | 128 | | 50 | 112 | 111 | 137 | 186 | 96 | 85 | 143 | 130 |
| MAN07 | 104 | 38 | 236 | | 83 | 95 | 85 | 106 | 92 | 105 | 89 | 95 | 105 |
| MAN08 | 71 | 99 | 63 | | 37 | 44 | 48 | 41 | 39 | 56 | 35 | 43 | 73 |
| MAN09 | 44 | 51 | 33 | | 26 | 30 | 29 | 31 | 25 | 29 | 27 | 28 | 72 |
| MAN10 | | 34 | 60 | 57 | 114 | | | | | | | | |

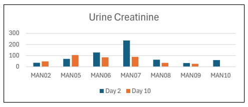


| Urine Albumin | Screening | Day 1 | Day 2 | Day 3** | Day 4 | Day 5 | Day 6 | Day 7 | Day 8 | Day 9 | Day 10 | Day 11 | Day 17 |
|---|---|---|---|---|---|---|---|---|---|---|---|---|---|
| MAN02 | 847.1 | 1185 | 829.2 | 1314 | 1700 | 1177 | 1153 | 617.6 | 1061 | 1135.2 | 1116 | 1710 | 1156 |
| MAN05* | 3776 | 2067 | 1084 | | 1088 | 553.3 | 1330 | 1088 | 1416 | 1499 | 2096.4 | 1908 | 607.9 |
| MAN06 | 5030 | 4933 | 1978 | | 661.6 | 2106 | 1283.5 | 1923 | 1783 | 1314 | 554.5 | 1525 | 994 |
| MAN07 | 3034 | 1227 | 5064 | | 1775.8 | 1737 | 1772.8 | 1895.8 | 1350 | 1716 | 1948 | 1712.3 | 1716 |
| MAN08 | 4654 | 4996 | 2101 | | 1819 | 1624 | 1966 | 1562 | 1475 | 2027 | 1470 | 1446 | 3136 |
| MAN09 | 2670 | 3117 | 1560 | | 1435 | 1101 | 777.1 | 1041.9 | 863 | 893 | 837.1 | 843 | 1607 |
| MAN10 | | 293.2 | 360.3 | 416.9 | 652.7 | | | | | | | | |

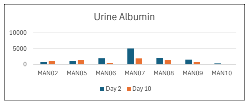


| Urine PCR | Screening | Day 1 | Day 2 | Day 3** | Day 4 | Day 5 | Day 6 | Day 7 | Day 8 | Day 9 | Day 10 | Day 11 | Day 17 |
|---|---|---|---|---|---|---|---|---|---|---|---|---|---|
| MAN02 | 3.545 | 3.511 | 3.028 | 4.14 | 2.986 | 3.568 | 3.762 | 4.095 | 4.897 | 3 | 2.917 | 4.907 | 3.333 |
| MAN05 | 4.207 | 2.844 | 2.479 | | 1.944 | 2.564 | 2.494 | 2.051 | 2.032 | 1.876 | 2.616 | 1.7 | 2.656 |
| MAN06 | 3.824 | 3.439 | 1.992 | | 1.84 | 2.223 | 1.784 | 1.679 | 1.387 | 1.833 | 1.035 | 1.566 | 1.046 |
| MAN07 | 3.442 | 3.974 | 2.758 | | 2.578 | 2.642 | 2.976 | 2.67 | 2.315 | 2.143 | 2.831 | 2.663 | 2.143 |
| MAN08 | 9.479 | 7.01 | 5.063 | | 9.027 | 5.818 | 5.729 | 5.488 | 5.795 | 5.054 | 5.457 | 5.163 | 6.233 |
| MAN09 | 8.659 | 9.941 | 8.485 | | 7.423 | 5.933 | 4.724 | 5.355 | 6 | 5.207 | 4.815 | 4.929 | 3.472 |
| MAN10 | | 1.294 | 0.933 | | 1.14 | 0.842 | | | | | | | |

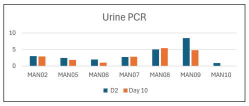


| Urine ACR | Screening | Day 1 | Day 2 | Day 3** | Day 4 | Day 5 | Day 6 | Day 7 | Day 8 | Day 9 | Day 10 | Day 11 | Day 17 |
|---|---|---|---|---|---|---|---|---|---|---|---|---|---|
| MAN02 | 2567 | 2521.3 | 2303.3 | 3055.8 | 2361.1 | 2675 | 2745.2 | 2941 | 3658.6 | 2141.9 | 2325 | 3976.7 | 2408.3 |
| MAN05 | 3120.7 | 1896.3 | 1526.8 | | 1208.9 | 1418.7 | 1683.5 | 1394.9 | 1506.4 | 1427.6 | 1677.1 | 1272 | 1899.7 |
| MAN06 | 2858 | 2212.1 | 1545.3 | | 1323.2 | 1880.4 | 1156.3 | 1403.6 | 958.6 | 1368.8 | 652.4 | 1066.4 | 764.6 |
| MAN07 | 2917.3 | 3228.9 | 2145.8 | | 2139.5 | 1828.4 | 2085.6 | 1788.5 | 1467.4 | 1634.3 | 2188.8 | 1802.4 | 1634.3 |
| MAN08 | 6554.9 | 5046.5 | 3334.9 | | 4916.2 | 3690.9 | 4095.8 | 3809.8 | 3782.1 | 3619.6 | 4200 | 3362.8 | 4295.9 |
| MAN09 | 6068.2 | 6111.8 | 4727.3 | | 5519.2 | 3670 | 2679.7 | 3361 | 3452 | 3079.3 | 3100.4 | 3010.7 | 2231.9 |
| MAN10 | | 862.4 | 600.3 | | 731.4 | 572.5 | | | | | | | |

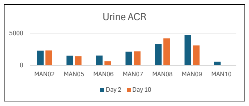


| Serum Creatinine | Screening | Day 1 | Day 2 | Day 3** | Day 4 | Day 5 | Day 6 | Day 7 | Day 8 | Day 9 | Day 10 | Day 11 | Day 17 |
|---|---|---|---|---|---|---|---|---|---|---|---|---|---|
| MAN02 | 1.28 | 1.35 | 1.19 | 1.31 | 1.19 | 1.33 | | 1.28 | 1.21 | | 1.31 | 1.16 | 1.49 |
| MAN05 | 2.75 | 2.91 | 2.95 | 3.01 | 2.95 | 3.06 | 3.16 | 3.1 | 3 | 3.14 | 3.43 | 3.37 | 3.13 |
| MAN06 | 0.84 | 0.79 | 0.9 | | 0.77 | 0.77 | | 0.91 | | | 0.88 | 0.88 | 0.96 |
| MAN07 | 1.32 | 1.2 | 1.24 | | 1.12 | 1.2 | 1.21 | 1.24 | 1.25 | 1.29 | 1.33 | 1.2 | 1.24 |
| MAN08 | 2.34 | 2.6 | 2.56 | | 2.33 | 2.3 | 2.11 | 2.34 | 2.21 | 2.51 | | 2.4 | 2.67 |
| MAN09 | 1.85 | 2.02 | 2.01 | 2.09 | 1.84 | 2.21 | 2.45 | 2.18 | 2.14 | 2.41 | 2.69 | 2.39 | 2.66 |
| MAN10 | | 1.35 | 1.21 | | 1.15 | 1.3 | | | | | | | |

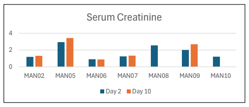


| eGFR | Screening | Day 1 | Day 2 | Day 3** | Day 4 | Day 5 | Day 6 | Day 7 | Day 8 | Day 9 | Day 10 | Day 11 | Day 17 |
|---|---|---|---|---|---|---|---|---|---|---|---|---|---|
| MAN02 | 61 | 57 | 67 | 60 | 67 | 58 | | 61 | 65 | | 59 | 69 | 51 |
| MAN05 | 29 | 29 | 28 | 28 | 28 | 27 | 26 | 27 | 28 | 26 | 24 | 24 | 26 |
| MAN06 | 82 | 89 | 76 | | 92 | 92 | | 75 | | | 78 | 78 | 70 |
| MAN07 | 79 | 89 | 86 | | 97 | 89 | 88 | 86 | 85 | 82 | 79 | 89 | 86 |
| MAN08 | 28 | 25 | 25 | | 28 | 29 | 32 | 28 | 30 | 26 | | 27 | 24 |
| MAN09 | 35 | 31 | 32 | 30 | 35 | 28 | 25 | 29 | 29 | 25 | 26 | 30 | 26 |
| MAN10 | | 56 | 64 | | 68 | 58 | | | | | | | |

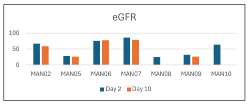


| Serum Albumin | Screening | Day 1 | Day 2 | Day 3** | Day 4 | Day 5 | Day 6 | Day 7 | Day 8 | Day 9 | Day 10 | Day 11 | Day 17 |
|---|---|---|---|---|---|---|---|---|---|---|---|---|---|
| MAN02 | 3.3 | 3.3 | 2.8 | 3.2 | 3.3 | 2.8 | | 2.6 | 2.7 | | 2.8 | 2.8 | 3.1 |
| MAN05 | 3.7 | 3.7 | 3.5 | | 3.3 | 3.6 | 3.3 | 3.6 | 3.3 | 3.4 | 3.8 | 3.5 | 3.7 |
| MAN06 | 3.5 | 3 | 3 | | 3.1 | 3 | | 3 | | | 3.2 | 3.1 | 3.4 |
| MAN07 | 3.6 | 3.4 | 3.2 | | 3 | 3.1 | 3.2 | 3.3 | 3.2 | 3.3 | 3.4 | 3.5 | 3.4 |
| MAN08 | 3.6 | 3.4 | 3.3 | | 3 | 2.7 | 3 | 3.1 | 3.1 | 3.3 | 3.1 | 3.4 | 3.6 |
| MAN09 | 3.6 | 3.7 | 3.2 | | 2.9 | 3.4 | 3.2 | 3 | 3.2 | 3.4 | 3.4 | 3.8 | 4.1 |
| MAN10 | | 3.5 | 3.5 | | 3.1 | 3.6 | | | | | | | |

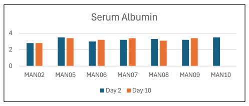


| Total cholesterol | Screening | Day 1 | Day 2 | Day 3** | Day 4 | Day 5 | Day 6 | Day 7 | Day 8 | Day 9 | Day 10 | Day 11 | Day 17 |
|---|---|---|---|---|---|---|---|---|---|---|---|---|---|
| MAN02 | 185 | 176 | 155 | 175 | 189 | 235 | | 206 | 222 | | 206 | 206 | 180 |
| MAN05 | 201 | 212 | 200 | | 191 | 194 | 188 | 190 | 172 | | | 177 | 219 |
| MAN06 | 249 | 208 | 208 | | 229 | 221 | | 222 | | | 223 | 222 | 206 |
| MAN07 | 295 | 330 | 324 | | 275 | 274 | | 267 | | | | 260 | 316 |
| MAN08 | 179 | 176 | 168 | | 153 | 141 | | 146 | | | | 158 | 175 |
| MAN09 | | 188 | 163 | | 134 | 153 | | 146 | | | | 173 | 147 |
| MAN10 | | 205 | 207 | | 173 | 186 | | | | | | | |

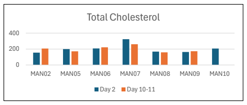


| Triglycerides | Screening | Day 1 | Day 2 | Day 3** | Day 4 | Day 5 | Day 6 | Day 7 | Day 8 | Day 9 | Day 10 | Day 11 | Day 17 |
|---|---|---|---|---|---|---|---|---|---|---|---|---|---|
| MAN02 | 116 | 93 | 71 | 111 | 79 | 154 | | 77 | 89 | | 114 | 86 | 138 |
| MAN05 | 231 | 209 | 218 | | 150 | 211 | 292 | 181 | 196 | | | 303 | 220 |
| MAN06 | 115 | 159 | 110 | | 154 | 140 | | 108 | | | 110 | 132 | 162 |
| MAN07 | 130 | 249 | 237 | | 132 | 153 | | 103 | | | | 102 | 210 |
| MAN08 | 181 | 234 | 190 | | 347 | 147 | | 174 | | | | 210 | 325 |
| MAN09 | | 244 | 135 | | 145 | 132 | | 118 | | | | 174 | 191 |
| MAN10 | | 317 | 304 | | 243 | 196 | | | | | | | |

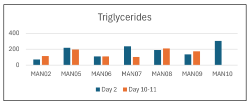


| HCT | Screening | Day 1 | Day 2 | Day 3** | Day 4 | Day 5 | Day 6 | Day 7 | Day 8 | Day 9 | Day 10 | Day 11 | Day 17 |
|---|---|---|---|---|---|---|---|---|---|---|---|---|---|
| MAN02 | 39.9 | 37.1 | 35.1 | 37.5 | 37.8 | 39.7 | | 37.8 | 40.2 | | 38.8 | 39.9 | 39.8 |
| MAN05 | 39.5 | 39.3 | 37.2 | 36.7 | | 32.1 | 33.2 | 34.3 | | 32.5 | 35.2 | 31.7 | 33.9 |
| MAN06 | 30.5 | 30.5 | 32.6 | | 32.3 | 31.4 | | 31.3 | | | 30.4 | 31.1 | 32.6 |
| MAN07 | 50.2 | 50.7 | 49.9 | | 46.3 | 48.1 | 47.3 | 48 | 47.7 | 49.4 | 48.5 | 49.3 | 48.9 |
| MAN08 | 38.2 | 35.8 | 37 | | 35.6 | 33.3 | 38.1 | 36.5 | 37.9 | 35.9 | | 35.9 | 37.5 |
| MAN09 | 39.9 | 39.2 | 34.5 | 37.8 | 31.4 | 34.4 | 32.5 | 31.8 | 33.2 | 34.4 | 32.3 | 36.1 | 38.4 |
| MAN10 | | 29.5 | 32 | | 29.8 | 31.1 | | | | | | | |

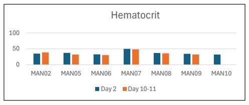


| Uric acid | Screening | Day 1 | Day 2 | Day 3** | Day 4 | Day 5 | Day 6 | Day 7 | Day 8 | Day 9 | Day 10 | Day 11 | Day 17 |
|---|---|---|---|---|---|---|---|---|---|---|---|---|---|
| MAN02 | 5.1 | 5.7 | 5.2 | 5.4 | 5.3 | 5.2 | | 5.4 | | | 4.9 | 5 | 6.1 |
| MAN05 | 6.9 | 7 | 6.5 | 6.3 | 6.1 | 6.6 | 6.2 | 6.4 | | 6.5 | | 6.4 | 8.1 |
| MAN06 | 8.9 | 8.5 | 8.4 | | 7.5 | 7.4 | | 7.7 | | | 8.8 | 8.8 | 9.9 |
| MAN07 | 8 | 7.3 | 6.3 | | 5.5 | 5.7 | | 5.9 | | | | 6.4 | 7.4 |
| MAN08 | 6.8 | 7.4 | 7.6 | | 7.8 | 7.6 | | 7.1 | | | | 6.9 | 7.8 |
| MAN09 | 9.2 | 8.5 | 8.4 | | 7.6 | 9 | | 10.9 | | | | 10.5 | 10.8 |
| MAN10 | | 9.9 | 9.8 | | 9.8 | 10.4 | | | | | | | |

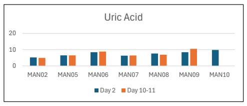


| Calcium | Screening | Day 1 | Day 2 | Day 3** | Day 4 | Day 5 | Day 6 | Day 7 | Day 8 | Day 9 | Day 10 | Day 11 | Day 17 |
|---|---|---|---|---|---|---|---|---|---|---|---|---|---|
| MAN02 | 2.34 | 2.17 | 2.15 | 2.17 | 2.2 | 2.12 | | 2.09 | 2.11 | | 2.07 | 2.15 | 2.12 |
| MAN05 | 2.27 | 2.3 | 2.25 | | 2.22 | 2.13 | 2.14 | 2.23 | 2.19 | 2.15 | 2.23 | 2.15 | 2.26 |
| MAN06 | 2.37 | 2.2 | 2.28 | | 2.25 | 2.23 | | 2.23 | | | 2.27 | 2.24 | 2.37 |
| MAN07 | 2.34 | 2.27 | 2.22 | | 2.09 | 2.21 | 2.22 | 2.2 | 2.14 | 2.23 | 2.27 | 2.24 | 2.24 |
| MAN08 | 2.42 | 2.38 | 2.38 | | 2.66 | 2.64 | 2.77 | 2.87 | 3.06 | 2.9 | 2.53 | 2.64 | 2.49 |
| MAN09 | 2.36 | 2.37 | 2.2 | | 1.89 | 2.19 | 2.25 | 2.12 | 2.29 | 2.29 | 2.26 | 2.46 | 2.46 |
| MAN10 | | 2.37 | 2.27 | | 2.18 | 2.3 | | | | | | | |

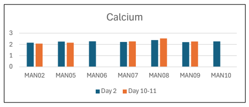


| Platelets | Screening | Day 1 | Day 2 | Day 3** | Day 4 | Day 5 | Day 6 | Day 7 | Day 8 | Day 9 | Day 10 | Day 11 | Day 17 |
|---|---|---|---|---|---|---|---|---|---|---|---|---|---|
| MAN02 | 260 | 278 | 238 | 259 | 290 | 229 | | 238 | 214 | | 258 | 238 | 273 |
| MAN05 | 220 | 222 | 198 | 188 | | 165 | 177 | 172 | 166 | 177 | 188 | 155 | 207 |
| MAN06 | 222 | 222 | 227 | | 227 | 209 | | 221 | | | 231 | 222 | 277 |
| MAN07 | 233 | 194 | 183 | | 161 | 160 | 152 | 167 | 161 | 182 | 186 | 184 | 205 |
| MAN08 | 258 | 286 | 269 | | 248 | 231 | 237 | 253 | 251 | 233 | | 243 | 271 |
| MAN09 | 397 | 384 | 324 | 360 | 289 | 311 | 321 | 318 | 349 | 386 | 386 | 452 | 452 |
| MAN10 | | 388 | 420 | | 355 | 404 | | | | | | | |

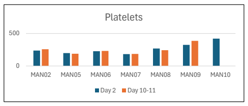


**Single Dose Phase (Day 2)**
**BID Dosing Phase (Day 5-9)**

****Day 3: Patients MAN02, MAN05, MAN10 received a single dose and were discharged.
Patients MAN02 and MAN05 returned for a separate in-patient visit at Day 3 or 4 for their BID dosing phase.
Patient MAN10 received only 1 single dose of 6 g ManNAc and stayed as inpatient until Day 5.**

***Patient MAN05 stopped dosing at Day 9 after evening dose and only received 4 days of BID dosing (instead of 5) bc of a perceived Adverse Event in the morning of Day 5.**

# VITAL SIGNS

**Temp**

| | Screen | Day 1 | Day 2 | Day 3 | Day 4 | Day 5 | Day 6 | Day 7 | Day 8 | Day 9 | Day 10 | Day 11 | Day 17 |
|---|---|---|---|---|---|---|---|---|---|---|---|---|---|
| MAN02 | 36 | | 35.9 | 35.8 | 35.9 | 36.7 | 36.1 | 36.7 | 36 | 36.6 | 36.4 | 37 | 35.8 |
| MAN05 | 36.3 | 36.5 | 36.7 | 36.2 | 36.5 | 36.1 | 35.8 | 36.2 | 36.3 | 36.1 | 36.7 | 36.8 | 36.9 |
| MAN06 | 37.3 | 37.5 | 36.8 | 36.5 | 36.9 | 36.1 | 36.2 | 36.6 | 36.2 | 36.1 | 36.2 | 36.7 | 37 |
| MAN07 | 36 | 36.6 | 36.6 | 35.4 | 35.8 | 36 | 35.7 | 35.9 | 35.4 | 35.7 | 36.8 | 36 | 36.7 |
| MAN08 | 36.9 | 36.8 | 36.6 | 36.5 | 36.4 | 36.9 | 36.8 | 36.5 | 35.8 | 36.3 | 35.7 | 36.5 | 36.6 |
| MAN09 | 36.5 | 36.6 | 35.6 | 35.7 | 36.5 | 36.1 | 36.4 | 36.6 | 36.6 | 36 | 36.1 | 36 | 35.9 |
| MAN10 | 36.9 | 37 | 36.3 | 37 | 36.8 | | | | | | | | |

**Pulse**

| | Screen | Day 1 | Day 2 | Day 3 | Day 4 | Day 5 | Day 6 | Day 7 | Day 8 | Day 9 | Day 10 | Day 11 | Day 17 |
|---|---|---|---|---|---|---|---|---|---|---|---|---|---|
| MAN02 | 62 | 59 | 57 | 61 | 56 | 63 | 69 | 60 | 64 | 64 | 61 | 67 | 60 |
| MAN05 | 50 | 59 | 62 | 51 | 64 | 59 | 65 | 68 | 61 | 66 | 73 | 68 | 82 |
| MAN06 | 85 | 100 | 82 | 85 | 76 | 80 | 83 | 76 | 99 | 88 | 75 | 80 | 116 |
| MAN07 | 77 | 81 | 80 | 62 | 69 | 78 | 77 | 87 | 81 | 84 | 75 | 71 | 81 |
| MAN08 | 76 | 78 | 91 | 62 | 60 | 54 | 61 | 57 | 67 | 74 | 70 | 69 | 73 |
| MAN09 | 55 | 55 | 46 | 50 | 58 | 68 | 71 | 67 | 74 | 66 | 69 | 73 | 70 |
| MAN10 | 91 | 77 | 75 | 81 | 82 | | | | | | | | |

**SBP**

| | Screen | Day 1 | Day 2 | Day 3 | Day 4 | Day 5 | Day 6 | Day 7 | Day 8 | Day 9 | Day 10 | Day 11 | Day 17 |
|---|---|---|---|---|---|---|---|---|---|---|---|---|---|
| MAN02 | 112 | 105 | 119 | 121 | 113 | 119 | 125 | 121 | 114 | 122 | 109 | 118 | 109 |
| MAN05 | 143 | 141 | 134 | 140 | 135 | 111 | 116 | 147 | 120 | 141 | 151 | 147 | 138 |
| MAN06 | 124 | 133 | 115 | 120 | 104 | 128 | 110 | 91 | 127 | 123 | 132 | 119 | 114 |
| MAN07 | 128 | 125 | 137 | 130 | 123 | 123 | 130 | 139 | 126 | 133 | 120 | 124 | 125 |
| MAN08 | 119 | 116 | 92 | 129 | 121 | 103 | 119 | 114 | 102 | 121 | 121 | 144 | 118 |
| MAN09 | 145 | 180 | 215 | 186 | 169 | 140 | 140 | 141 | 136 | 146 | 128 | 147 | 151 |
| MAN10 | 153 | 125 | 136 | 134 | 119 | | | | | | | | |

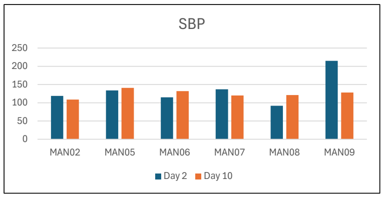


**DBP**

| | Screen | Day 1 | Day 2 | Day 3 | Day 4 | Day 5 | Day 6 | Day 7 | Day 8 | Day 9 | Day 10 | Day 11 | Day 17 |
|---|---|---|---|---|---|---|---|---|---|---|---|---|---|
| MAN02 | 61 | 72 | 78 | 77 | 79 | 80 | 83 | 70 | 81 | 79 | 72 | 75 | 58 |
| MAN05 | 82 | 71 | 77 | 77 | 59 | 43 | 56 | 68 | 42 | 66 | 75 | 68 | 85 |
| MAN06 | 71 | 74 | 61 | 73 | 66 | 75 | 63 | 50 | 68 | 78 | 73 | 63 | 63 |
| MAN07 | 81 | 85 | 95 | 89 | 84 | 79 | 77 | 79 | 72 | 77 | 82 | 85 | 77 |
| MAN08 | 72 | 63 | 53 | 68 | 64 | 63 | 69 | 59 | 72 | 75 | 72 | 69 | 67 |
| MAN09 | 58 | 61 | 82 | 57 | 54 | 67 | 57 | 56 | 65 | 64 | 61 | 65 | 55 |
| MAN10 | 88 | 63 | 68 | 70 | 61 | | | | | | | | |

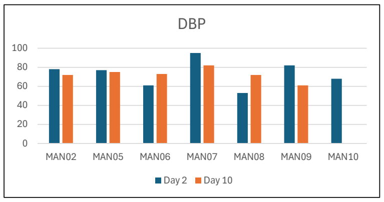


**Weight**

| | Screen | Day 1 | Day 2 | Day 3 | Day 4 | Day 5 | Day 6 | Day 7 | Day 8 | Day 9 | Day 10 | Day 11 | Day 17 |
|---|---|---|---|---|---|---|---|---|---|---|---|---|---|
| MAN02 | 70.2 | 70.7 | | | | 73.8 | | | | | | | 75.2 |
| MAN05 | 135.5 | 135.6 | | | | 141 | | | 139.2 | | 138.4 | | 140.3 |
| MAN06 | 87.9 | 87.5 | 87 | | | | 85.8 | 85.6 | 85.4 | 85.6 | 84.9 | | 85.5 |
| MAN07 | 103.1 | 104.9 | | 104.8 | 105.6 | 105.6 | 105.4 | 105.3 | 106 | 107.1 | 107.2 | 106.9 | 105.34 |
| MAN08 | 139.4 | 138 | 136.8 | 136.3 | 136.3 | 136.4 | 136 | 136.1 | 136.3 | 136.1 | 136.9 | 136.6 | 136.4 |
| MAN09 | 90 | 89.2 | 87.8 | 87.1 | 87.7 | 87 | 85.1 | 87.5 | 86.8 | 86.5 | 87 | 86.8 | 88 |
| MAN10 | 147.3 | 144.8 | 144.3 | 143.1 | 143.1 | | | | | | | | |

**Day 2: Single dose**

**Day 5-9: BID dosing**

# ManNAc PK

| | | Single Dose (SD) Phase | | | | | | | | Wash-out after SD | | | BID dosing phase | | | | | | | | | Follow-up Phase | | | |
|---|---|---|---|---|---|---|---|---|---|---|---|---|---|---|---|---|---|---|---|---|---|---|---|---|---|
| | Day 1 | Day 2 | | | | | | | | Day 3 | | Day 4 | Day 5 | | | Day 6 | Day 7 | Day 8 | Day 9 | | | Day 10 | | Day 11 | Day 17 |
| | BL | 0h | 0.5h | 1h | 2h | 4h | 6h | 8h | 12h | 24h | 36h | 48h | 72h SD t0h BID | BID non-fasting | t0 BID | t24h BID | t48h BID | t72h BID | MAN05 stopped ManNAc | t96h BID | t108h last dose; t0 post | t12h post BID | t24h post BID | t36h post BID | 1 week post |
| MAN02 | 57.9 | 48.6 | 451 | 926 | 1660 | 1370 | 857 | 521 | 219 | 70.4 | 54.6 | 46.6 | 55.6 | 57 | 60.2 | 294 | 419 | 427 | | 324 | 301 | 411 | 65.9 | 57.9 | 89.8 |
| MAN05 | 96.8 | 95.3 | 692 | 1250 | 1550 | 1500 | 1010 | 798 | 449 | 136 | 114 | 96.3 | 96.9 | 141 | 85.7 | 639 | 527 | 643 | 1280 | 248 | 179 | 170 | 163 | 156 | 163 |
| MAN06 | 47.0 | 42.0 | 646 | 1220 | 1970 | 2130 | 1520 | 916 | 330 | 88.2 | 113 | 43.5 | 78.4 | | | 296 | 329 | 412 | | 296 | 240 | 353 | 104 | 85.6 | 68.6 |
| MAN07 | 61.6 | 40.7 | 330 | 799 | 1130 | 1170 | 748 | 385 | 97.7 | 44.0 | 35.9 | 33.6 | 30.7 | | | 151 | 118 | 263 | | 159 | 75.3 | 85.4 | 33.6 | 26.8 | 48.3 |
| MAN08 | 106 | 103 | 816 | 1820 | 3680 | 4690 | 3470 | 2970 | 1700 | 281 | 136 | 108 | 110 | | | 1020 | 1120 | 1710 | | | 853 | 1220 | 1190 | 134 | 109 |
| MAN09 | 138 | 89 | 289 | 615 | 852 | 1580 | 1280 | 1210 | 929 | 269 | 146 | 92 | 130 | | | 1290 | 1320 | 1350 | | 735 | 737 | 940 | 355 | 204 | 112 |
| MAN10 | 40.5 | 57 | 472 | 1010 | 1040 | 782 | 431 | 333 | 176 | 46.7 | 42.9 | 34.1 | 41.3 | | | | | | | | | | | | |

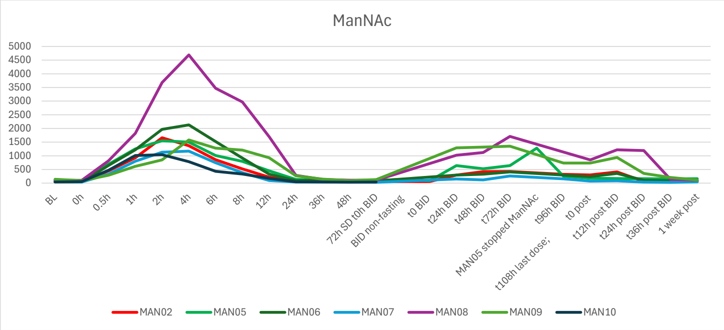


**Day 5:** Patients MAN02 and MAN05 received a single dose first and were discharged. They returned for BID dosing on a separate visit and a PK blood draw was performed the evening they were re-admitted - the day before Day 5 dosing (BID non-fasting).

MAN10 only received 1 single dose (6 g ManNAc)

Patient MAN05 stopped dosing at Day 9 (no morning + evening dose) and only received 4 days of BID dosing (instead of 5) bc of a perceived Adverse Event in the morning of Day 5

# Neu5Ac PK

| | non-fasting | Single Dose (SD) phase | | | | | | | | no dosing/washout | | | BID dosing phase | | | | | | | | | Follow-up Phase | | | |
|---|---|---|---|---|---|---|---|---|---|---|---|---|---|---|---|---|---|---|---|---|---|---|---|---|---|
| | Day 1 | Day 2 | | | | | | | | Day 3 | | Day 4 | Day 5 | | | Day 6 | Day 7 | Day 8 | Day 9 | | | Day 10 | | Day 11 | Day 17 |
| | Screen | 0h | 0.5h | 1h | 2h | 4h | 6h | 8h | 12h | 24h | 36h | 48h | 72h (t0) | BID non-fasting | t0 BID | t24h BID | t48h BID | t72h BID | MAN05 stopped ManNAc | t96h BID | t108h last dose t0 post | t12h post BID | t24h post BID | t36h post BID | 1 week post |
| MAN02 | 345 | 307 | 265 | 291 | 301 | 410 | 463 | 491 | 446 | 393 | 353 | 328 | 350 | 380 | 322 | 656 | 967 | 1020 | | 913 | 1390 | 1070 | 968 | 590 | 382 |
| MAN05 | 603 | 597 | 605 | 521 | 562 | 845 | 915 | 1080 | #### | 864 | 646 | 693 | 621 | 633 | 645 | 1230 | 1630 | 1780 | 1760 | 1480 | 1250 | 1150 | 888 | 897 | 614 |
| MAN06 | 310 | 285 | 278 | 317 | 331 | 476 | 506 | 566 | | 372 | 433 | 325 | 343 | | | 714 | 931 | 1120 | | 997 | 1820 | 926 | 1060 | 486 | 368 |
| MAN07 | 369 | 359 | 370 | 372 | 384 | 559 | 683 | 706 | 494 | 385 | 394 | 356 | 340 | | | 530 | 694 | 721 | | 590 | 695 | 586 | 509 | 423 | 379 |
| MAN08 | 819 | 732 | 718 | 768 | 799 | 1040 | 1420 | 1510 | #### | 1540 | 1410 | 1070 | 846 | | | 2180 | 3000 | 3600 | | | 3880 | 4550 | 3380 | 2190 | 926 |
| MAN09 | 629 | 620 | 601 | 603 | 608 | 711 | 795 | 901 | 993 | 1010 | 968 | 664 | 773 | | | 1560 | 1850 | 1950 | | 2420 | 2590 | 2070 | 2450 | 1600 | 1030 |
| MAN10 | 415 | 344 | 308 | 342 | 377 | 388 | 443 | 469 | 437 | 381 | 362 | 320 | 350 | | | | | | | | | | | | |

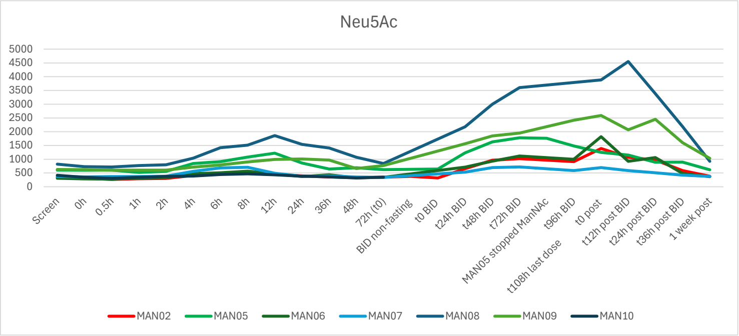


**Day 5:** Patients MAN02 and MAN05 received a single dose first and were discharged. They returned for BID dosing on a separate visit and a PK blood draw was performed the evening they were re-admitted - the day before Day 5 dosing (BID non-fasting).

MAN10 only received 1 single dose (6 g ManNAc)

Patient MAN05 stopped dosing at Day 9 (no morning + evening dose) and only received 4 days of BID dosing (instead of 5) bc of a perceived Adverse Event in the morning of Day 5

## Table S3: Participant Concomitant Medications[1]

| Subject | Steroid use pattern | IS at BL | Non-specific anti-proteinurics at BL |
|---|---|---|---|
| ***Cohort A*** | | | |
| MAN02 | SD, PR | - | Lisinopril |
| MAN05 | Never on steroids[2] | - | Losartan |
| MAN06 | Never on steroids for renal disease[3] | - | Losartan |
| MAN07 | SR | P | Telmisartan |
| MAN08 | Steroid Intolerant | - | Lisinopril |
| MAN09 | SR | - | Lisinopril, Spironolactone |
| ***Cohort B*** | | | |
| MAN10 | SD,PR | M,T,P | Lisinopril |

*Abbreviations*: BL, baseline; IS, Immunosuppressive therapy; M, Mycophenolate mofetil; P, Prednisone; PR, partial response; SD, Steroid dependent; SR, Steroid Resistant; T, Tacrolimus.

[1]The potential for *ManNAc* (or *SA)* to interfere with the absorption of concurrent therapies remains unknown and warrants evaluation in future clinical trials. In contrast, the impact of ongoing therapies on ManNAc absorption appears minimal. Pharmacokinetic (PK) profiles following a single ManNAc dose, including time to maximum plasma concentration (Tmax) and clearance, were consistent across participants receiving various background medications. These PK profiles closely match data from prior studies involving individuals not receiving concomitant medications, suggesting that the listed background treatments may have limited impact on ManNAc bioavailability.

[2]Subjects MAN05 never received immunosuppression.

[3]Subject MAN06 declined steroid therapy for renal disease following a prior history of steroid use for ulcerative colitis, which has resulted in significant side effects. This earlier treatment occurred several years before the diagnosis of FSGS.

# Table S4: Mixed Effects Repeated Measures Model Estimates

| **Mixed Effects Repeated Measures Model Estimates** [1] *UPCR mean of study Days 1&2 versus Days 10&11* | | | | | | | | |
|---|---|---|---|---|---|---|---|---|
| | **Mean estimate of % change** | **Mean Estimate** Confidence Limits | | **Random Effects** | ***p*-value** | **Base-line $log_{10}$ uPCR variance** [2] | **EBLUP range limits** [3] | |
| Estimate, as modeled for this study[4] | -9.69% | -13.65% | -5.54% | Intercepts | <0.0001 | 0.01013 | -0.1738 | 0.1524 |

[1] Models employing only post-screening timepoint-assessed values, and excluding patient MAN10 (who only received a single ManNAc dose).

[2] Baseline Neu5Ac and eGFR ‘adjusted estimates’ mixed effects repeated measures model of $log_{10}$-UPCR over time.

[3] Empirical Best Linear Unbiased Prediction (EBLUP) range limits for baseline UPCR random intercepts’ deviation from overall (across-individuals mean) intercept.

[4] Mixed-effects model commensurate to available statistical information in this phase 1 study modeling $log_{10}$-UPCR linear in time with a random intercept for each participant adjusted for baseline UPCR, Neu5Ac, and Day 2 eGFR (66 repeated measures across 6 participants, **Supplementary Table S2**).

# Supplementary Methods: Statistical Analysis

## 1: Mixed Effects Repeated Measures Model Estimates

We employed a specific instance of mixed-effects models for repeated measures (MMRM), a modeling approach utilized broadly across therapeutic development trials since the International Conference on Harmonisation adopted the estimand framework within its Efficacy Principle E9 Revision 1 Addendum (https://www.ich.org/page/efficacy-guidelines#9). Our MMRM instance, once leveraging approximate linearity on the log-transformed scale for UPCR repeated measures, judiciously spent its modest degrees of freedom on common-across-individuals intercept and slope as fixed effects. The actual data had modest degrees of freedom, given this early-phase trial's short treatment and follow-up timeframe constraint on the number of repeated measures and its limited enrollment size. We looked to accommodate the anticipated heterogeneity in log-transformed UPCR levels (especially given use of spot rather than 24-hour urine samples) via a random effect varying by participant, or 'random intercept'.

## 2: Linear-by-linear and Jonckheere-Terpstra association test

The linear-by-linear (LxL) association test is appropriate when assessing the extent to which two ordered-value variables are associated with one another [Agresti et al. J Am Stat Assoc 1990 (Ref #41)]. This test accommodates *a priori* weighting schemes, here chosen to be the quantitative ordered values for UPCR and equally-spaced ordinal scores for degree of (hypo)sialylation, while an alternate rank-based option for hypothesis testing (Jonckheere-Terpstra) will be similarly applied to assess robustness of LxL findings given use of spot-urine values subject to measurement error relative to a 24-hour "gold-standard" measurement [Sahu et al. Indian J Clin Biochem 2022 (Ref #51)].

It is effectively applied to the cell-specific counts that result from cross-tabulating observations in terms of two such variables (e.g., rows for one ordered-value variable, columns for the other). As proteinuria reduction for each participant results in a unique value (ordered relative to that of other individuals' values), such a cross-tabulation of this with biopsy-assessed degree of glomerular hyposialylation yields *sparse* cell counts of zero or one, we employed an *exact* version of the linear-by-linear association test and did so considering a single direction of association (i.e., a linear rank test whose reference distribution is exactly determined by all possible permutations of row-by-column table counts that match the *observed* cross-tabulation's marginal totals). This ensures that, in contrast to a test that leverages large-sample approximations as a reference distribution when making inference, the operating characteristics of *our* association test (such as significance level, or maximum acceptable rate of false positive conclusions under the null hypothesis of no association) can be maintained under modest sample size and sparse counts. Employing such a test with interest in the alternative hypothesis of positive association, our inference is that the degree of proteinuria reduction appears associated with the degree of glomerular hyposialylation (exact linear-by-linear association test at 5% significance level).

These findings warrant validation via reproducing a consistent conclusion in similar follow-on studies. Notably, due to *a priori* scientific considerations as outlined under Discussion, this inference applies only when considering one-sided positively correlated trends and leveraging observed changes in UPCR values as linear ranks; this was not a prior-specified hypothesis test so should be considered hypothesis-generating. For this reason, we also present here a set of auxiliary findings available through the *un*weighted rank-based alternate test for doubly-ordered cross-tabulated sparse counts, the Jonckheere-Terpstra (JT) linear-rank test. As some information is lost relative to the LxL association test and this is corroborative yet complementary to its inferences, we weight each participants' contributions to the JT test by the corresponding number of glomeruli assessed for their biopsy. Additionally, rather than using the coarsened Normal/Moderate/Severe trichotomy, we use the estimated percent (of controls' mean) sialylation, instead, as the exact form of the test can accommodate sparse (0/1) counts. This yields a one-sided p-value for the exact test of 0.0016 (directional as for the LxL test), and a two-sided p-value of 0.0032. Future trials may explore proteinuria-change/hyposialylation association using approaches such as these, regardless of how modestly sized their designs may be.

## Supplementary Methods: Glomerular Sialylation Analysis

*Kidney Biopsy Slides*

Unstained formalin-fixed, paraffin-embedded sections from a (diagnostic) kidney biopsy taken prior to this study were obtained for each subject, under procedures adherent to the Declaration of Helsinki. The diagnosis of each patient was confirmed by the pathology reports received with each biopsy. The original pathology report for MAN02 was missing, so the biopsy date is unknown; the FSGS diagnosis of MAN02 was confirmed by NIH pathology on pre-study biopsy slides. Anonymized control adult kidney tissue was obtained either from the NIDDK, NIH collection (Ctr#1: AK6), or from autopsy tissues acquired from the National Disease Research Interchange (NDRI), Philadelphia (Ctr#2: OD21568 and Ctr#3: OD23129). Due to limited availability of kidney tissue and the explorative nature of this study, 2 unstained slides for lectin histology were acquired per biopsy.

*Fluorescence Lectin and Antibody Staining*

The biopsy sections (5 µm) of all 7 subjects and 3 controls were subjected to lectin histochemistry in one batch under the same conditions, as previously described [Kakani et al. Am J Pathol 2012 (Ref#7); Niethamer et al. Mol Genet Metab 2012 (Ref #9)]. Briefly, the sections were deparaffinized in Hemo-De solvent (Scientific Safety Solvents, Keller, TX, USA) and rehydrated, followed by antigen retrieval (heating in 0.01 mol/L sodium citrate, pH 6.4 solution) and blocking in Carbo-Free blocking solution (Vector Laboratories, Newark, CA, USA). All slides were then double-stained by incubation with 10 µg/mL FITC-SNA lectin [Iskratsch et al. Anal Biochem 2009 (Ref #39)] (elderberry bark agglutinin from *Sambucus nigra*, predominantly binding terminal α[2,6]-linked SA [Neu5Ac] endgroups on glycans; #FL1301-2, Vector Laboratories) and a monoclonal antibody against the podocyte membrane marker CD151 (#H-8, sc-271216, Santa Cruz Biotechnology, Santa Cruz, CA, USA) used in 1:10 dilution in Carbo-Free blocking solution overnight at 4°C. After washing (0.1% Triton X-100 in 1X Tris-buffered saline (TBS) for 3 times, followed by a wash in 1X TBS), the slides were incubated with secondary antibodies (to visualize the CD151 staining) donkey-anti-mouse AlexaFluor555 (Thermo Fisher Scientific, Waltham, MA, USA) in Carbo-Free Blocking solution at room temperature for 1 hour. After washing again (0.1% Triton X-100 in 1XTBS for 3 times, followed by 1X TBS), all slides were incubated in 0.3% Sudan black for 10 minutes to reduce autofluorescence, washed again (1X TBS) and mounted with Vectashield containing the nuclear dye 4′,6-diamidine-2′-phenylindole dihydrochloride (DAPI, Vector Laboratories).

*Confocal Microscopy and Image Analysis*

Imaging of all sections was performed in one batch with the same microscope settings. All glomeruli (if present) on each biopsy slide were digitally imaged in Z-stacks with a 40X oil DIC objective on a Zeiss LSM880 confocal laser-scanning microscope (Carl Zeiss Microscopy, Jena, Germany). Fluorescent images were created from each collapsed Z-stack (1-D projections, as shown in **Figure 4A)**, and 7-slices from the center of each Z-stack were used for fluorescence quantification with Maximum Intensity Projection using Zen 2.1 SP3 software (Carl Zeiss Microscopy). For this quantification, the 7-slice stacks of all glomeruli on each slide were manually outlined as ROI (**Figure 4A**) and the software's histogram function was used for fluorescence intensity quantitation of the SNA and CD151 signals in each ROI. An area of minimum intensity inside each ROI was used for background corrections. Average fluorescence intensity of SNA (*green*) divided by the average fluorescence intensity of CD151 (*red*) in co-localized areas within each ROI were determined and reported for each glomerulus. The number of glomeruli per slide are indicated in **Figure 4C**; note that the biopsy slides from patients MAN05 and MAN10 contained no glomeruli and were not informative for this analysis. The SNA/CD151 fluorescence intensity of all glomeruli in all 3 control slides combined (19 total glomeruli) were averaged and arbitrarily set as 100% sialyation. The average SNA signal/CD151 signal of all glomeruli per patient slide was then determined, including its percentage from the control slides. We defined severe hyposialylation (*red* highlight in Table below) in glomeruli that

have SNA-determined sialylation of less than 50% of normal, moderate hyposialylation (*orange*) between 50-85% of normal, and normal sialylation (*green*) in levels of more than 85% of normal sialylation.

## Glomerular Fluorescence Intensity Data

| Subject # | # Glomeruli | SNA/CD151 | % glomerular sialylation | Sialylation group |
|---|---|---|---|---|
| Ctr#1 | 7 | 0.6168 | - | |
| Ctr#2 | 6 | 0.8091 | - | |
| Ctr#3 | 6 | 0.8706 | - | |
| Ave Ctrs | 19 | 0.75766 | 100% | Normal sialylation |
| MAN02 | 7 | 0.96609 | 128% | Normal sialylation |
| MAN05 | 0 | - | - | - |
| MAN06 | 6 | 0.56356 | 74% | Moderate hyposialylation |
| MAN07 | 6 | 0.48831 | 64% | Moderate hyposialylation |
| MAN08 | 6 | 0.54922 | 72% | Moderate hyposialylation |
| MAN09 | 5 | 0.26740 | 35% | Severe hyposialylation |
| MAN10 | 0 | - | - | - |

| Section/topic | No | CONSORT 2025 checklist item description | Reported on page no. |
|---|---|---|---|
| **Title and abstract** | | | |
| Title and structured abstract | 1a | Identification as a randomised trial – Our trial was non-randomized single arm (open label) | NA |
| | 1b | Structured summary of the trial design, methods, results, and conclusions | 2 |
| **Open science** | | | |
| Trial registration | 2 | Name of trial registry, identifying number (with URL) and date of registration | https://www.clinicaltrials.gov/study/NCT02639260 (7/7/2016) |
| Protocol and statistical analysis plan | 3 | Where the trial protocol and statistical analysis plan can be accessed | Corresponding author and/or NIDDK upon request; manuscript Supplementary Methods |
| Data sharing | 4 | Where and how the individual de-identified participant data (including data dictionary), statistical code and any other materials can be accessed | NIDDK upon request |
| Funding and conflicts of interest | 5a | Sources of funding and other support (eg, supply of drugs), and role of funders in the design, conduct, analysis and reporting of the trial | 18-19 |
| | 5b | Financial and other conflicts of interest of the manuscript authors | 18 |
| **Introduction** | | | |
| Background and rationale | 6 | Scientific background and rationale | 4-5 |
| Objectives | 7 | Specific objectives related to benefits and harms | 5-8 |
| **Methods** | | | |
| Patient and public involvement | 8 | Details of patient or public involvement in the design, conduct and reporting of the trial | 18-19 |
| Trial design | 9 | Description of trial design including type of trial (eg, parallel group, crossover), allocation ratio, and framework (eg, superiority, equivalence, non-inferiority, exploratory) | 5-8 |
| Changes to trial protocol | 10 | Important changes to the trial after it commenced including any outcomes or analyses that were not prespecified, with reason | NA |
| Trial setting | 11 | Settings (eg, community, hospital) and locations (eg, countries, sites) where the trial was conducted | 5 |
| Eligibility criteria | 12a | Eligibility criteria for participants | Suppl Table S1 |
| | 12b | If applicable, eligibility criteria for sites and for individuals delivering the interventions (eg, surgeons, physiotherapists) | NA |
| Intervention and comparator | 13 | Intervention and comparator with sufficient details to allow replication. If relevant, where additional materials describing the intervention and comparator (eg, intervention manual) can be accessed | 5-8 |
| Outcomes | 14 | Prespecified primary and secondary outcomes, including the specific measurement variable (eg, systolic blood pressure), analysis metric (eg, change from baseline, final value, time to event), method of aggregation (eg, median, proportion), and time point for each outcome | 7-8 |
| Harms | 15 | How harms were defined and assessed (eg, systematically, non-systematically) | 7-8 |
| Sample size | 16a | How sample size was determined, including all assumptions supporting the sample size calculation | 7 |
| | 16b | Explanation of any interim analyses and stopping guidelines | NA |
| Randomisation: | | | |
| Sequence generation | 17a | Who generated the random allocation sequence and the method used | NA |
| | 17b | Type of randomisation and details of any restriction (eg, stratification, blocking and block size) | NA |

| | | | Reported on page no. |
|---|---|---|---|
| Allocation concealment mechanism | 18 | Mechanism used to implement the random allocation sequence (eg, central computer/telephone; sequentially numbered, opaque, sealed containers), describing any steps to conceal the sequence until interventions were assigned | NA |
| Implementation | 19 | Whether the personnel who enrolled and those who assigned participants to the interventions had access to the random allocation sequence | NA |
| Blinding | 20a | Who was blinded after assignment to interventions (eg, participants, care providers, outcome assessors, data analysts) | NA |
| | 20b | If blinded, how blinding was achieved and description of the similarity of interventions | NA |
| Statistical methods | 21a | Statistical methods used to compare groups for primary and secondary outcomes, including harms | Suppl Statistical Methods |
| | 21b | Definition of who is included in each analysis (eg, all randomised participants), and in which group | NA |
| | 21c | How missing data were handled in the analysis | NA |
| | 21d | Methods for any additional analyses (eg, subgroup and sensitivity analyses), distinguishing prespecified from post hoc | Suppl Statistical Methods |
| **Results** | | | |
| Participant flow, including flow diagram | 22a | For each group, the numbers of participants who were randomly assigned, received intended intervention, and were analysed for the primary outcome | NA |
| | 22b | For each group, losses and exclusions after randomisation, together with reasons | Figure 2 |
| Recruitment | 23a | Dates defining the periods of recruitment and follow-up for outcomes of benefits and harms | Figure 2, Page 9 |
| | 23b | If relevant, why the trial ended or was stopped | Figure 2, Page 9 |
| Intervention and comparator delivery | 24a | Intervention and comparator as they were actually administered (eg, where appropriate, who delivered the intervention/comparator, how participants adhered, whether they were delivered as intended (fidelity)) | NA |
| | 24b | Concomitant care received during the trial for each group | Suppl Table S3, Page 6, Page 9 |
| Baseline data | 25 | A table showing baseline demographic and clinical characteristics for each group | Table 1 |
| Numbers analysed, outcomes and estimation | 26 | For each primary and secondary outcome, by group:<br>● the number of participants included in the analysis<br>● the number of participants with available data at the outcome time point<br>● result for each group, and the estimated effect size and its precision (such as 95% confidence interval)<br>● for binary outcomes, presentation of both absolute and relative effect size | Suppl Methods, Suppl Table S4, Pages 9-13, 15-16 |
| Harms | 27 | All harms or unintended events in each group | Table 3 |
| Ancillary analyses | 28 | Any other analyses performed, including subgroup and sensitivity analyses, distinguishing pre-specified from post hoc | NA |
| **Discussion** | | | |
| Interpretation | 29 | Interpretation consistent with results, balancing benefits and harms, and considering other relevant evidence | 13-17 |

| | | | |
|---|---|---|---|
| Limitations | 30 | Trial limitations, addressing sources of potential bias, imprecision, generalisability, and, if relevant, multiplicity of analyses | 16-17 |

Citation: Hopewell S, Chan AW, Collins GS, Hróbjartsson A, Moher D, Schulz KF, et al. CONSORT 2025 Statement: updated guideline for reporting randomised trials. BMJ. 2025; 388:e081123. https://dx.doi.org/10.1136/bmj-2024-081123
© 2025 Hopewell et al. This is an Open Access article distributed under the terms of the Creative Commons Attribution License (https://creativecommons.org/licenses/by/4.0/), which permits unrestricted use, distribution, and reproduction in any medium, provided the original work is properly cited.

*We strongly recommend reading this statement in conjunction with the CONSORT 2025 Explanation and Elaboration and/or the CONSORT 2025 Expanded Checklist for important clarifications on all the items. We also recommend reading relevant CONSORT extensions. See www.consort-spirit.org.
